# Green Metallic Nanoparticles: Biosynthesis to Applications

**DOI:** 10.3389/fbioe.2022.874742

**Published:** 2022-04-06

**Authors:** Hitesh Chopra, Shabana Bibi, Inderbir Singh, Mohammad Mehedi Hasan, Muhammad Saad Khan, Qudsia Yousafi, Atif Amin Baig, Md. Mominur Rahman, Fahadul Islam, Talha Bin Emran, Simona Cavalu

**Affiliations:** ^1^ Chitkara College of Pharmacy, Chitkara University, Rajpura, India; ^2^ Yunnan Herbal Laboratory, College of Ecology and Environmental Sciences, Yunnan University, Kunming, China; ^3^ The International Joint Research Center for Sustainable Utilization of Cordyceps Bioresources in China and Southeast Asia, Yunnan University, Kunming, China; ^4^ Department of Biochemistry and Molecular Biology, Faculty of Life Science, Mawlana Bhashani Science and Technology University, Tangail, Bangladesh; ^5^ Department of Biosciences, COMSATS University Islamabad, Sahiwal, Pakistan; ^6^ Unit of Biochemistry, Faculty of Medicine, University Sultan Zainal Abidin, Kuala Terengganu, Malaysia; ^7^ Department of Pharmacy, Faculty of Allied Health Sciences, Daffodil International University, Dhaka, Bangladesh; ^8^ Department of Pharmacy, BGC Trust University Bangladesh, Chittagong, Bangladesh; ^9^ Faculty of Medicine and Pharmacy, University of Oradea, Oradea, Romania

**Keywords:** nanoparticle, green nanotechnology, preparation, synthesis, application

## Abstract

Current advancements in nanotechnology and nanoscience have resulted in new nanomaterials, which may pose health and environmental risks. Furthermore, several researchers are working to optimize ecologically friendly procedures for creating metal and metal oxide nanoparticles. The primary goal is to decrease the adverse effects of synthetic processes, their accompanying chemicals, and the resulting complexes. Utilizing various biomaterials for nanoparticle preparation is a beneficial approach in green nanotechnology. Furthermore, using the biological qualities of nature through a variety of activities is an excellent way to achieve this goal. Algae, plants, bacteria, and fungus have been employed to make energy-efficient, low-cost, and nontoxic metallic nanoparticles in the last few decades. Despite the environmental advantages of using green chemistry-based biological synthesis over traditional methods as discussed in this article, there are some unresolved issues such as particle size and shape consistency, reproducibility of the synthesis process, and understanding of the mechanisms involved in producing metallic nanoparticles *via* biological entities. Consequently, there is a need for further research to analyze and comprehend the real biological synthesis-dependent processes. This is currently an untapped hot research topic that required more investment to properly leverage the green manufacturing of metallic nanoparticles through living entities. The review covers such green methods of synthesizing nanoparticles and their utilization in the scientific world.

## Introduction

To lessen the risks associated with nanotechnology, the ideal option is to use green nanotechnology in manufacturing and implementation. One of the most significant advancements in nanotechnology and materials science is the creation of engineered nanomaterials (Mu et al., 2021; Sun et al., 2021). Nanotechnology has penetrated various fields such as drug delivery and other biomedical applications (Chopra et al., 2021a, 2021b, 2022; Singla et al., 2021; Bhattacharya et al., 2022). Moving these things out of the lab and into the real world is the only way to bring them to life. There are tens of thousands of these goods on the market, most found in daily personal care, cosmetics, and apparel. Commercializing successful breakthrough technologies is essential for a wide range of human applications and worldwide progress, but critical attention is required in the materials’ potential, health evaluation, and environmental impact. There’s little doubt that nanoparticles (NPs) provide a health concern that has to be handled quickly, and their production and use are essentially unregulated, especially in the development of nanoscale materials. While new chemical processes are designed with little risk in mind, hazardous compounds are minimized or eliminated by applying the foundational principles of green chemistry. This is a crucial feature of the green chemistry developing industry (Hassan et al., 2021).

A large amount of time and effort has been devoted to developing acceptable synthetic methods for creating nanoparticles because of their physiochemical characteristics and many uses. However, environmental contamination produced by heavy metals restricts several physiochemical techniques to form metal nanoparticles. As a result, the manufacturing of nanoparticles by biological methods has emerged as a new trend in the industry due to its nontoxicity, repeatability, ease of scaling up, and well-defined shape. Researchers have found that novel resources such as microbes and plants have the most potential for producing nanoparticles (Shafey, 2020; Lahiri et al., 2021; Cuong et al., 2022; Devra, 2022; Ettadili et al., 2022; Lomelí-Rosales et al., 2022; Majeed et al., 2022; Mustapha et al., 2022; Najafi et al., 2022). Metal nanoparticles have been synthesized using a variety of microorganisms, including bacteria, fungus, and yeast, as well as plants. “Green synthesis” is necessary to prevent the generation of undesirable or dangerous by-products *via* the build-up of dependable, sustainable, and eco-friendly synthesis techniques. The usage of optimal solvent systems and natural resources (such as organic systems) is vital to attain this aim. Green production of metallic nanoparticles has been utilized to accommodate diverse biological components (e.g., bacteria, fungi, algae, and plant extracts). Among the current greenways of synthesis for metal/metal oxide nanoparticles, the use of plant extracts is a straight forward technique to generate nanoparticles at large scale in comparison to bacteria and/or fungal assisted synthesis. These compounds are known together as biogenic nanoparticles. Here, we present an update on recent breakthroughs in the synthesis of biological nanoparticles and outline their future development and potential uses (Abu Hajleh et al., 2021).

## Green Synthesis of Nanoparticles

In recent studies, it has been proven that microorganisms and plants may be used to synthesize nanoparticles in a way that is both ecologically friendly and safe to use (Makarov et al., 2014; Gowramma et al., 2015). Microbes and plants have always been able to collect and store inorganic metallic ions from their environment. Because of their enticing properties, many living organisms have effective biological factories, minimizing pollution while also recovering metals from industrial waste. The capacity of a living organism to employ its metabolic processes to transform inorganic metallic ions into metal nanoparticles has opened the door to a relatively new and primarily untapped area of study (Baker et al., 2013). Since discovering microbes’ ability to interact with, remove, and gather metallic elements from their surroundings, several biotechnological applications, such as bioremediation and bioleaching have been developed. They can interact with their environment because of their lipid-based amphipathic membranes, which allow for various oxidation-reduction events to take place and promote biochemical transformations (Cavalu et al., 2020). Microorganisms grown in specific settings may also accelerate linked oxidation and reduction in nanoparticle formation (Belliveau et al., 1987; Kowshik et al., 2002; Durán et al., 2005; Lengke et al., 2006). Still, the oxidation-reduction mechanisms are unknown to humans. Much research is still needed to fully understand and explain the differences in nanoparticle size and form across different metals when they are created by the same microorganism (Bhattacharya and Gupta, 2005). Even when it comes to using plants to make nanoparticles, this is still true. There are several advantages to using plants instead of other ecologically friendly biological systems like bacteria and fungi, such as eliminating expensive and time-consuming preparation and isolation methods. Contrastingly, the use of plants or plant-derived extracts to create nanoparticles is usually regarded as safer and more efficient than the use of other biological systems for nanoparticle production. Another advantage of plant-based biosynthesis over different ways is that it is a straightforward process that can readily be scaled up for the large-scale manufacture of nanoparticles. This is a significant advantage over other alternatives.

Nanoparticle production is possible with each living organism’s specific biochemical processing abilities. Nanoparticles can only be synthesized by certain biological organisms because of their enzyme activity and metabolic processes. As a result, to produce nanoparticles with well-defined features such as size and form, it is necessary to carefully pick the appropriate biological entity. However, there are a few exceptions to the general rule that biological entities with a high capacity for heavy metal accumulation are more likely to synthesize metallic nanoparticles. When working with microorganisms, the methods used to cultivate them are essential. Many culturing parameters, including nutrition, light intensity, medium pH, temperature, mixing speed, and buffer strength, must be optimized to increase enzyme activity (Singh et al., 2010). An innovative alternative to standard chemical synthesis and the more challenging growth and isolation techniques necessary for many microorganisms has recently been discovered in the biological creation of nanoparticles using plants and plant extracts. A combination of compounds found in plant extracts has been shown to reduce and stabilize (cap) the formation of nanoparticles (Narayanan and Sakthivel, 2008; Sathishkumar et al., 2009; Singh et al., 2010). As a result of their complexity and non-toxicity, these biological molecules have become more popular.

### Biosynthesis of Nanoparticles Using Plants

**FIGURE 1:**
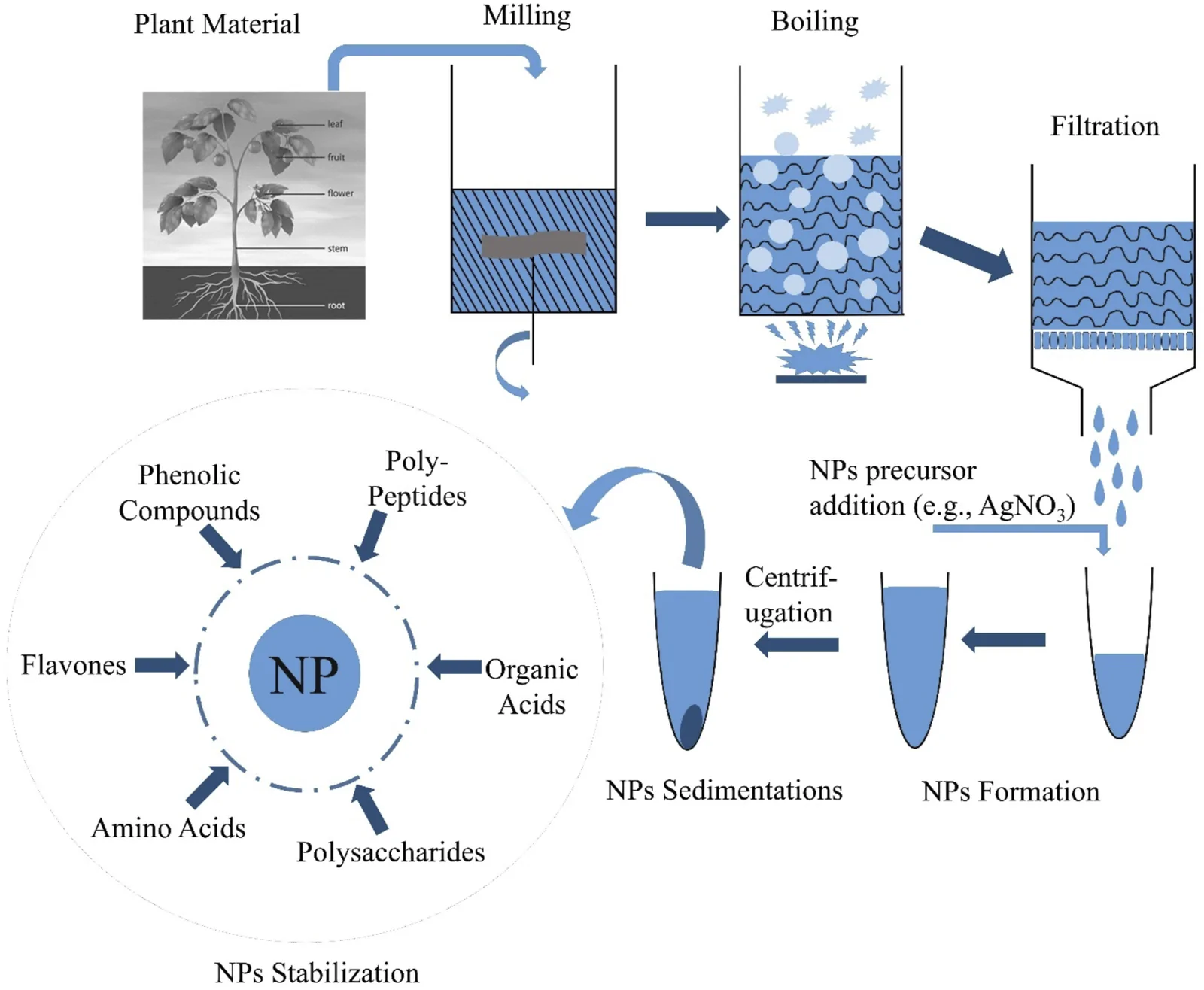
Schematic representation of biosynthesis of nanoparticles from plants.

Plant nanotechnology has recently opened up new pathways for the production of nanoparticles and is an environmentally benign, simple, quick, and stable technique. Using water as the solvent to synthesize nanoparticles has several benefits, including biocompatibility, scalability, and medicinal application (Noruzi, 2015). This means that plant-derived nanoparticles may meet the rising demand for nanoparticles with applications in biomedicine and the environment since they are made from easily accessible plant components and are not hazardous. Gold and silver nanoparticle synthesis utilizing *Panax ginseng* leaf and root extract has recently been shown to be possible using medicinal plants as sources of raw materials (Singh et al., 2016c; 2016b). In addition, metal nanoparticles have been synthesized using different plant components, such as the leaves, fruits, and stems, and their extracts. The pathway for biosynthesis of nanoparticles from plants has been shown in Figure 1.

Furthermore, it has been suggested that secondary metabolites such as flavonoids and alkaloids have important functions in metal salt reduction and capping and stabilizing agents for nanoparticles generated from proteins and amino acids (Duan et al., 2015). *Corallina officinalis* extract, for example, contains polyphenols and proteins with carbonyl groups that might help create and stabilize gold nanoparticles (El-Kassas and El-Sheekh, 2014). Silver and gold nanoparticles were synthesized and stabilized in *Murraya koenigii* leaf extract by Philip et al. (Philip et al., 2011).

According to the literature review, biologically synthesized NPs are more active than physicochemically synthesized NPs. Metallic NPs produced from plant extracts are stable and mono-dispersible when the pH, incubation time, mixing ratio, and temperature are all accurately regulated. Curry, mango, neem, turmeric, and guava have all been utilized to create Gold NPs. Plant extracts are rich in polyphenols, which hasten the breakdown of organic materials (Huston et al., 2021). Metal NPs may be harvested from plants with a great capacity to decrease metal ions both on their surface and in multiple organs and tissues distant from the ion penetration point. According to the metal bioaccumulation study, nanoparticles (NPs) are the most frequent metal deposit (Khalaj et al., 2020). If you look at extracts from plants and look for compounds like terpenoids and phenolic acids (Rahman et al., 2021c) as well as proteins in spectroscopic measurements, you’ll see that metal ions may be reduced to nanostructured forms. Shankar SS et al. employed geranium leaf extract to synthesize silver NPs extra-cellularly using the rapid reduction of silver ions in an aqueous silver nitrate solution. In solution, the particles produced quasilinear superstructures ranging in size from 16 to 40 nm, which were seen by transmission electron microscopy (TEM) and found to be very stable and crystalline (Shankar et al., 2003).

By allowing reduction processes to run using aqueous solutions of AgNO3 and chloroauric acid, researchers could produce pure metallic and bimetallic silver and gold nanoparticles using the broth of Neem (*Azadirachta indica*) leaves. During the examination, silver and gold nanoparticles were polydispersed and flat plate-like in shape. After being exposed to Au3+ and Ag + ions in solution, bimetallic Au core-Ag shell NPs were formed, which were verified by TEM analysis and further showed that the Ag NPs served as adsorbents onto the gold NPs, resulting in the core-shell structure (Shankar et al., 2004). Using *Murraya koenigii* leaf extract, Laura Christen and others were able to produce silver nanoparticles as part of their investigation on the impact of broth concentration on the reduction process and particle size. As previously mentioned, the broth was removed in the same way. Leaf broth content was investigated using reaction mixtures comprising 1:25, 1:50, 1:100, and 1:200 AgNO_3_ with 1:100 leaf broth and 1:250 leaf broth to 10^−3^ M AgNO_3_. The reduction process was investigated using UV analysis. At 435 nm, the absorbance peak was recorded at a range beyond the normal range (Christensen et al., 2011). The researchers found that the reduction rate and particle size decreased along with the agglomeration tendency when broth content increased.

FTIR investigations have indicated that polyphenols in green tea extract may be used as a capping agent as well as a reducer, as proven by researchers (Gottikumala, 2017). Satoaki Onitsuka and others synthesized gold and silver nanoparticles (NPs) from the Camellia sinensis plant extracts. Precursors such as HAuCl4 or AgNO3 were used to react with the tea leaf extracts as catalysts (aqueous solutions). At low temperatures in ambient conditions, HAuCl4 or AgNO_3_ aqueous solutions were combined with aqueous NaHCO_3_ to create metal NP solutions, which were then tested. It has been revealed that the antimicrobial pigments included in Ag and Au NPs have diameters of 30 and 10 nm, respectively (Onitsuka et al., 2019).

In 2017, Roy created silver nanoparticles using neem leaf extracts (Roy et al., 2017). AgNO3 and neem extract solution was also stored at a low light level in a dark room as a precautionary measure. Silver nanoparticles showed antibacterial efficacy against *E. coli* and Gram-positive bacteria but were more effective than either. The UV absorption peak was discovered to be between 420 and 450 nm. According to the study’s results, Ag NPs may be produced at a lower concentration of plant extract (Rahman et al., 2021b) (Roy et al., 2017). In the production of silver NPs, the leaves of *Azadirachta indica* and Triphala have been employed. Neem leaves yielded Ag NPs of 43 nm in diameter, whereas Triphala leaves yielded 59 nm-diameter Triphala-derived Ag NPs. Neem and Triphala were shown to reduce the growth of gentamicin and ampicillin-resistant *K. pneuomoniae*, and similar findings were seen in *S. Typhi* resistant to gentamicin and piperacillin (Gavhane et al., 2012).

*Calotropis procera*, a member of the *Asclepiadaceous* family, was used to synthesize CuO NPs sustainably (Reddy, 2017). Because of their low bandgap, these NPs are frequently employed in numerous applications such as photocatalysis (Li J. et al., 2011). It is utilized to treat splenic, pilescausal, and tumor-related disorders. Fresh Calotropis leaves were cleaned in distilled water and dried in the Sun before being chopped into fine pieces and put in deionized water to boil until the solution became yellow, as described in this article. It was then added to the solution with cupric acid and heated until a blue-green paste was formed. At 700°C, this powder was calcined till the substance’s color became black.

By employing Punica granatum peels extract, Alaa Y. Ghidan et al. were able to green manufacture copper nanoparticles (Ghidan et al., 2016). *Punica granatum’s* fresh peels are picked and washed numerous times to eliminate any contaminants before being used in cooking. It was discovered that the color of the solution changed from white to yellow when the powdered peels were blended with distilled, sterile water and then boiled. CuO NPs were produced by dissolving copper acetate powder in water and stirring it with a magnetic stirrer for some time. It was discovered that after adding the *P. granatum* extract, the solution changed colorfrom green to brown, suggesting the formation of monodispersed Cu NPs in the solution.

Copper NPs were freshly synthesized from fresh *Abutilon indicum* leaves by Ijaz et al. (Ijaz et al., 2017). They were gently washed to remove dust and dry and shadowed sections from the fresh *Abutilon indicum* leaves. The leaves were ground up and sieved through a 200-nm screen to make fuel.

*Abutilon indicum* extract, Copper (II) nitrate trihydrate, and double-distilled water made CuO NPs. The solution was homogenized for 2–5 min using a magnetic stirrer. Next, a pre-heated muffle furnace was utilized to execute a combustion reaction on the mixture to yield CuO NPs at a temperature of 400 °C. The ash component of the plant extracts was removed by filtering the resulting combination. Methanol was used to eliminate contaminants from the solution after rinsing with distilled water. Researchers reported the synthesis of copper nanoparticles supported on sodium borosilicate glass using extract of Acalypha indica L. (Nasrollahzadeh et al., 2018b). The nanoparticles were able to reduce the 2,4-dinitrophenilhydrazine (2,4-DNPH), 4-nitrophenol (4-NP), methyl orange (MO), methylene blue (MB) and congo red (CR) by using NaBH_4_ in aqueous medium. Similarly copper nanoparticles synthesized from *Plantago asiatica* leaf extract, were reported to catalyze the direct cyanation of aldehyde using K_4_Fe(CN)_6_ (Nasrollahzadeh et al., 2017b).

Copper nanoparticles, were also reported to synthesize from, *Centella asiatica* L. leaf extract (Nasrollahzadeh et al., 2018c). The copper nanoparticles were further immobilized on the surface of manganese dioxide. The resulting nanoparticles were able to form the recycle catalyst that can be applied for the reduction of 2,4-DNPH and MB. Another, group of researchers prepared copper nanoparticles, using extracts of *Euphorbia maculata* aerial parts and reported their activity to reduce MB and RhB (Pakzad et al., 2019).

The bio-agent used in the synthesis of biological NPs significantly impacts the technology used to execute it. If you want to make metal NPs from plant extracts, for example, you’ll first need to gather, wash, and dry the plant component you want (such as a leaf or fruit), then crush it before extracting the resulting crystals from the solvent (Mohamad et al., 2014). Following this, the extract is filtered or centrifuged to remove the solid plant residue, and the metal precursor chemical is combined with the residue under certain circumstances. Several factors may affect the reaction time (and hence the properties of nanoparticles), such as the ratio of reactant concentrations and the temperature of the reaction, pH, light, ultrasound, or microwave heating (Varma, 2012; Mittal et al., 2013). Incubation is followed by high-speed centrifugation of the nanoparticles, which are entirely rinsed in water/solvent (e.g., ethanol, methanol) and collected (Singh et al., 2016d). The reaction conditions significantly impact the number and morphology (shape, size distribution) of the resultant nanoparticles. Additionally, the extracted source has a considerable impact since different plant extracts might range significantly in their concentration and mix of reducing and stabilizing biomolecules (Kumar and Yadav, 2009). Other plants contributing to the nanoparticle synthesis have been enlisted in Table 1.

**TABLE 1:** Reported data of plants responsible for nanoparticle synthesis.

Types of Nanoparticle	Plants	Size (nm)	Activity	Refs
Ag	*Red ginseng*	10–30	Antimicrobial activity against *Vibrio parahaemolyticus*, *Staphylococcus aureus, Bacillus cereus, and Candida albicans*	Singh et al. (2016a)
	*Azadirachta indica*	41–60	Larvicidal activity against *Aedes aegypti* and *Culex quinquefasciatus*	Poopathi et al. (2015)
	*Nigella sativa*	15	Less cytotoxic and phytotoxic in comparison to wet-chemistry synthesized nanoparticles	Amooaghaie et al. (2015)
	*Pistacia atlantica*	27	Antibacterial action against *S. aureus*	Sadeghi et al. (2015)
	*Nyctanthes arbortristis*	—	Antibacterial action against *Escherichia coli* MTCC 443	Gogoi et al. (2015)
	*Anogeissus latifolia*	5.5–5.9	Antibacterial action against Gram-positive and negative bacteria	Kora et al. (2012)
	*Pinus densiflora*	30–80	Antibacterial action against *B. linens, P. acnes, B. cereus,* and *S. epidermidis*	Velmurugan et al. (2015)
	*Acalypha indica*	20–30 nm; spherical	Antibacterial action against *E. coli* and *V. cholerae*	Krishnaraj et al. (2010)
	*Allium sativum (garlic clove)*	4–22 nm; spherical	Biocompatible to human lung epithelial A549 cell	Ahamed et al. (2011)
	*Catharanthus roseus*	48–67 nm	—	Kannan et al. (2011)
	*Citrus sinensis peel*	35 ± 2 nm (at 25°C), 10 ± 1 nm (at 60°C); spherical	Antibacterial action against *Escherichia coli*, *Pseudomonas aeruginosa*, and *Staphylococcus aureus*	Kaviya et al. (2011)
	*Coleus aromaticus*	44 nm	Antibacterial action against *Bacillus subtilis* and *Klebsiella planticola*	Vanaja and Annadurai, (2013)
	*Curcuma longa*	—	Antibacterial action against *E.coli*	Sathishkumar et al. (2010)
	*Eclipta prostrate*	35–60 nm; triangles, pentagons, hexagons	Larvicidal action against filariasis vector, *Culex quinquefasciatus say* and malaria vector, *Anopheles subpictus Grassi*	Rajakumar and Abdul Rahuman, (2011)
	Euphorbiaceae* latex*	18 nm Ag	Antibacterial action against *Escherichia coli* (*Gram-negative*), *Corney bacterium* (Gram-positive), *Bacillus substilus* (spore-forming)	Patil et al. (2012)
	*Eryngium planum*	26–42 nm	Inhibitory action against *S. aureus* and *B. subtilis*	Dehghan et al. (2022)
	*Garcinia mangostana (mangosteen leaf)*	35 nm	Antibacterial action against *E. coli* and *S. Aureus*	Veerasamy et al. (2011)
	*Gelidiella acerosa*	22 nm	Antifungal action against *Humicola insolens* (MTCC 4520), *Fusarium dimerum* (MTCC 6583)*, Mucor indicus* (MTCC 3318) and *Trichoderma reesei* (MTCC 3929)	Vivek et al. (2011)
	*Melia azedarach*	—	AgNPs synthesized showed superior cytotoxic activity compared to the *M. azedarach* aqueous extract	Sukirtha et al. (2012)
	*Moringa oleifera*	57 nm	Antibacterial action against *Staphylococcus aureus*, *Candida tropicalis*, *Candida* krusei, *Klebsiella pneumoniae*	Prasad and Elumalai, (2011)
	*Musa paradisiacal*	20 nm	Antibacterial action against *E. coli*, *E. aerogenes*, *Klebsiella* sp. and *Shigella* sp. and antifungal activity against *C. albicans* and *C. lipolytica*	Bankar et al. (2010)
	*Lonicera japonica*	-	Ag NPs damaged the morphology of A549 human lung cancer cells at the very lowest concentration	Rajivgandhi et al. (2022)
	*Cissus quadrangularis*	24 nm	Anti-arthritic activity	Kanimozhi et al. (2022)
	*Ziziphus Mauritiana*	10–45 nm	Anti-hepatic cancer activity	Sameem et al. (2022)
	*Conocarpus Lancifolius*	21–173 nm	Antiproliferative efficacy in the MDA-MB-231 breast cancer cell line by inducing apoptosis	Oves et al. (2022)
	*Striga angustifolia*	6.99 nm	Anti-microbial, antioxidant, and anti-proliferative activity in apoptotic p53 signaling pathway	Raja et al. (2022)
	*Carissa carandas*	33–37 nm	Anti-microbial action against *Shigella flexineri*	Bouafia et al. (2021)
	*Nelumbo nucifera (lotus)*	25–80 nm; spherical, triangular	Larvicidal action against *Anopheles subpictus Grassi* and *Culex quinquefasciatus Say*	Santhoshkumar et al. (2011)
Au	*Cymbopogon citratus*	20–50	Larvicidal action against *Anopheles subpictus Grassi* and *Culex quinquefasciatus Say*	Murugan et al. (2015)
	*Spinacia oleracea L*	16.7 nm	Anti-endometrial cancer activities against common endometrial cancer cell lines i.e., HEC-1-B, HEC-1-A, KLE, and Ishikawa	Zhu et al. (2022)
	*Tecoma capensis*	10–35 nm	Anticancer activity against MCF7 cancer cells	Hosny et al. (2022)
	*Jatropha integerrima Jacq*	38.8 nm	Antibacterial action against *B. subtilis, S. aureus, E. coli*, and *K. pneumoniae*	Suriyakala et al. (2022)
Cu	*Ginkgo biloba*	15–20	—	Nasrollahzadeh and Mohammad Sajadi, (2015)
	*Euphorbia bungei*	—	The catalyst for direct cyanation of aldehydes with K_4_ [Fe(CN)_6_]	Nasrollahzadeh et al. (2017a)
	*Euphorbia falcata*	The catalyst	Catalyst for the synthesis of amino- and N-sulfonyl tetrazoles	Motahharifar et al. (2020)
Pd	*Electro*	5–10	Electrocatalytics activity towards H_2_O_2_	Momeni and Nabipour, (2015)
	*Catharanthus roseus*	40	Photocatalytic degradation of Phenol red	Kalaiselvi et al. (2015)
	*Euphorbia thymifolia L*	—	—	Nasrollahzadeh and Sajadi, (2016)
	*Hibiscus tiliaceus*	—	Reductive catalysis of Cr(VI) and nitro compounds	Nasrollahzadeh et al. (2018a)
Lead	*Cocos nucifera*	47	Antibacterial action against *Staphylococcus aureusEscherichtalyticeria coli, Staphylococcus epidermis* and *Bacillus subtilis*	Elango and Roopan, (2015)
Titanium	*Euphorbia heteradena Jaub*	—	Catalytic activity for the Huisgen [3 + 2] cycloaddition of azides and alkynes	Nasrollahzadeh and Sajadi, (2015)
Fe_3_O_4_	*Punica granatum L*	21–23 nm	Optoelectronic application	Bouafia et al. (2021)

### Fungi Assisted Nanoparticle Synthesis

Fungal biomass and associated metabolites are used to synthesize NPs in a relatively young field of nanotechnology known as “myconanotechnology” (Gade et al., 2010). Micro- and macrofungi alike have several reducing enzymes and proteins, which provide a significant advantage in the production of NP. As opposed to bacteria, fungi generate a diverse spectrum of enzymes, which allows the transformation of metal salts into NPs to occur very quickly in contrast.

The bio-potential of the fungal cell wall is also assumed to be significant in the absorption and reduction of metal ions and the generation of metal NPs (Gade et al., 2010; Khan et al., 2018). It's still unclear precisely how NPs are generated or what biological components are involved in the process. It is suggested that fungus-mediated NPs arise either *in vivo* or *in vitro*. Most of the harmful transition metal ions are converted to a non-toxic form in the mycelia of the fungus during the *in vivo* method, which exploits this process to make NPs intracellularly. It is possible to directly use washed mushroom mycelia to produce NPs intracellularly in this method. Mycelia must undergo additional treatment to remove the NPs from the mycelia before being used again (Molnár et al., 2018).

In contrast, there are three approaches to make NPs from fungal cell-free extracts using the *in vitro* methodology. The first technique to produce NPs is to use the fermented fungus’s supernatant, which contains extracellular proteins and enzymes (Vágó et al., 2016). According to the second approach (Siddiqi and Husen, 2016). Bioengineered nanoparticles (e.g., AuNPs) may be generated by the intracellular components released into the medium due to the breakdown of cell walls. The use of fungal mycelia’s aqueous extract for the production of NPs is also an option. NPs may be produced by the autolysis of fungal cells, followed by the dissolution of membrane proteins and surface carbohydrates in the solution. The washing and re-suspension of fungal mycelia in a pathogen-free environment is a difficult and not always possible operation using this technique, as previously indicated (Kitching et al., 2016).

Most well-known NPs syntheses use Basidiomycota to produce edible mushrooms (Aygün et al., 2020). *Oyster* and *Ganoderma* species have been the subject of several studies in the last few years to learn more about the synthesis of NPs. The mushrooms produced through pure culture are non-pathogenic, non-toxic, and can create a wide variety of physiologically active proteins (Chopra et al., 2021c). Consequently, their higher than normal enzyme activity [108] can transform dangerous compounds into more minor toxic forms, create large amounts of biomass, and accumulate NPs in their mycelia and culture media. Since the discovery of NP synthesis by basidiomycetes, compared to the discovery of NP synthesis by other lower fungi and bacteria, more research into the process of NP synthesis is required.

As a result of its high bioactivity, *Ganoderma* sp. is one of the most investigated mushrooms for the production of NPs. Over 250 different varieties of this fungus have been identified. This includes *G. lucidum, G. applanatum,* G. capense, and *G. tsugae*. Pharmacological evaluation of *Ganoderma* sp. has demonstrated its antibacterial, anti-HIV, anti-inflammatory, anti-proliferative, anti-diabetic, anticancer, hypocholesterolemic, and hepato-protective potential (Sudheer et al., 2019; Mominur Rahman et al., 2021; Rahman et al., 2022). These mushrooms have also been proven to be helpful in the synthesis of NPs, particularly AgNPs. There are certain drawbacks when using mushrooms to synthesize metallic NPs, such as the need to maintain aseptic growing conditions, the possibility of contamination in samples, and the variability in NP size when using these mushrooms (Li X. et al., 2011). AgNPs were produced using *G. sessiliforme* and showed significant antibacterial and antioxidant activity (Mohanta et al., 2018). There are two prominent mushrooms, *G. lucidum* and *G. applanatum*, recognized for their bioactive components and antibacterial qualities in the culinary and medical worlds. To synthesize AgNPs, the mushrooms’ extracts were employed (Poudel et al., 2017; Al-Ansari et al., 2020).

There is evidence that an edible fungus called *Volvariella volvacea’s* aqueous extract contains one of the most effective agents for decreasing and capping the extracellular production of gold and silver nanoparticles (Au–Ag NPs) (Philip, 2009; Bhattacharya et al., 2022). Another long-term investigation used the immobilized fungus *Coriolus versicolor* to bioremediate cadmium salt and synthesize stable CdS nanoparticles in aqueous settings. Similar results were obtained. The immobilized fungus was shown to remove more than 90% of cadmium within 2 hours, whereas auto capped CdS NPs were produced under aqueous conditions, thereby providing a dual role in this investigation (Li X. et al., 2011).

The basidiomycete’s fungus was principally responsible for the production of AuNPs. The *P. citrinopileatus,* the *P. eous,* the *P. cystidiosus*, the *P. ostreatus*, *P. eryngii*, and the *P. flabellatus* were all examined. Tests for *Pulmonarius* were carried out as well (Madhanraj et al., 2017). Researchers found that the antioxidant properties of AuNPs synthesized by *P. Pulmonarius* were at their highest levels in the samples examined in antioxidant studies. Most AuNPs were synthesized from *P. eous* and *P. florida*, which had the most remarkable ability to reduce ferric oxide. A human colon cancer cell line, HCT-116, was tested with the AuNPs produced using *Lentinus sajor-caju (Fr.)* extract as the reducing agent (Chaturvedi et al., 2020). HCT-116s antiproliferative effects were dose-dependent and obvious right away. When the cancer cells were examined, they showed signs of cell integrity loss and DNA breakage.

To make AuNPs, researchers employed laccase extracted from *P. ostreatus* mushrooms (El-Batal et al., 2015). A greater amount of AuNPs was produced after the mushroom had been subjected to 5 kGy of gamma radiation. A radiolytic reduction is thought to have happened, in which radiolysis of the aqueous solution produced H_3_O+, H, OH, and H_2_O_2_ species, all of which reduced the metal salts into ionized form. *Flammulina velutipes*, a fungus, have also been demonstrated to produce AuNPs inside cells (Narayanan et al., 2015). The mycelium’s inner cell membrane was the primary location where AuNPs were found, which was not a surprise. An enzyme located on the inner surface of the cell membrane was thought to be responsible for reducing the gold precursors. Using phenolic compounds extracted from *G. applanatum*, an AuNP-based decolorization technique for methylene blue dye was developed recently, and the findings were reported in Nano Letters. An average of 18.7 nm-sized face-centered cubics AuNPs, capable of decolorizing methylene blue in 35 s, were spotted by our researchers using phenol-capped AuNPs (Abdul-Hadi et al., 2020). Another study found that *A. bisporus*-derived AuNPs with a size range of 10–50 nm had remarkable antifungal activity against the pathogenic fungus A. flavus (Eskandari-Nojedehi et al., 2018). Many other fungi responsible for synthesizing metallic nanoparticles have been enlisted in Table 2.

**TABLE 2:** Reported representation of fungus synthesizing silver nanoparticles.

Species	Size (nm)	Activity	Ref
Ag			
*Aspergillus fumigatus*	5–25	—	Bhainsa and D’Souza, (2006)
*Cryptococcus laurentii*	35–400	Antifungal action against *Botrytis cinerea* (BNM 0528), *Penicillium expansum* (CEREMIC 151–2002), *Aspergillus niger* (NRRL 1419), *Alternaria* sp. (NRRL 6410), and *Rhizopu* sp. (NRRL 695)	Fernández et al. (2016)
*Fusarium oxysporum*	5–13	Antibacterial activity against *Escherichia coli* and *Staphylococcus aureus*	Husseiny et al. (2015)
*Rhodotorula glutinis*	15–220	*Botrytis cinerea* (BNM 0528), *Penicillium expansum* (CEREMIC 151–2002), *Aspergillus niger* (NRRL 1419), *Alternaria* sp. (NRRL 6410), and *Rhizopus* sp. (NRRL 695)	Fernández et al. (2016)
*Letendraea* sp. *WZ07*	33.8 nm	Antibacterial activities against Gram-negative and Gram-positive bacteria	Qiao et al. (2022)
*P. ostreatus*	100 nm	Antibacterial activity against *S. aureus*	Mirunalini et al. (2012)
*P. ostreatus*	4–15 nm	Anticancer activity on MCF-7 cell line (breast carcinoma)	Yehia and Al-Sheikh, (2014)
*P. ostreatus*	40 nm	*Antibacterial activity against Bacillus subtilis*, *Bacillus cereus*, *Staphylococcus aureus, Escherichia coli,* and *Pseudomonas aeruginosa*	Al-Bahrani et al. (2017)
*P. florida*	20 nm	Antibacterial activity against *Staphylococcus aureus, Salmonella typhi, Providencia alcalifaciens,* and *Proteus mirabilis*	Bhat et al. (2011)
*P. florida*	N. D	Heterogeneous catalyst in the reduction of 4-nitrophenol (4-NP) to 4-aminophenol (4-AP)	Sen et al. (2013)
*P. cornucopiae *var.* citrinopileatus*	20–30 nm	Antifungal action against *Candida sp*	Owaid et al. (2015)
*P. citrinopileatus*	7 nm	—	Bhardwaj et al. (2018)
*P. sajor caju*	16.8 nm	Antifungal action against *Candida albicans*	Musa et al. (2018)
*P. djamor *var.* roseus*	5–50 nm	Cytotoxic against PC3 cells	Raman et al. (2015)
*Aspergillus sydowii*	5–15 nm	Antiproliferative activity to HeLa cells and MCF-7 cells *in vitro*	Wang et al. (2021)
*Aspergillus fumigatus KIBGE-IB33*	<100 nm	Anti-microbial activity against *Enterococcus faecalis* ATCC 29212	Raza et al. (2021)
*Fusarium oxysporum-NFW16*	32.7 nm	Synergistic effect with both vancomycin and ciprofloxacin against MRSA (25%), *Pseudomonas aeruginosa* (50%), and pus isolated *Escherichia coli* (50%)	Ilahi et al. (2021)

### Bacterial Mediated Synthesis

Critical metals must permeate the cell wall into the cytoplasm and then return through the meshwork of the cell wall to be discharged into the environment. Since the cell wall’s peptidoglycans provide polyanions for the metal-to-chemical reactive group stoichiometric interaction, metal is deposited on the cell wall in an inorganic form. Many metal-binding sites on the wall may be changed using chemical methods to convert positive charge to negative charge (an essential step in the metal-binding process). Chemical change of peptidoglycan of *Bacillus subtilis* allowed for improved metal penetration and deposition with a large quantity of metal (Beveridge and Murray, 1980). A factor of 20,000 to 40,000 above the extracellular concentration of metals may be found in the cell. Bacteria may benefit from dipole moments created by metal deposition by aligning themselves with the geomagnetic field. A cell’s internal and exterior environments and bacteria species with different morphologies frequently influence the crystalline and non-crystalline phases of particle formation when particles are created.

In the silver-resistant bacterial strain, *Pseudomonas stutzeri AG259,* which was isolated from a silver mine, internal accumulation of silver NPs, as well as some silver sulphide, with sizes ranging from 35 to 46 nm, was observed (Slawson et al., 1992). When *P. stutzeri AG259* was exposed to high concentrations of silver ions during growth, larger particles were produced, resulting in the intracellular synthesis of silver NPs ranging in size from a few nm to 200 nm (Klaus-Joerger et al., 2001). Silver detoxification was accomplished by the bacteria *P. stutzeri AG259* through precipitation in the periplasmic space and bioreduction to elemental silver with a variety of crystal typologies, including hexagons and equilateral triangles, as well as three different types of particles: elemental crystalline silver, monoclinic silver sulphide (Ag2S), and a further undetermined structure (Klaus et al., 1999). The thickness of the crystals was regulated by the periplasmic space, but not their width, which might be rather large (100–200 nm) due to the presence of the periplasmic space.

Psychrophilic bacteria *Phaeocystis antarctica*, *Pseudomonas meridiana, Arthrobacter kerguelensis,* Arthrobacter gangotriensis, and two mesophilic bacteria, *Bacillus* indicus, and *Bacillus cecembensis*, were employed to biosynthesize silver nanoparticles (NPs) with sizes ranging from 6 to 13 nm. These NPs stayed steady in the dark over 8 months. The generation and stability of silver nanoparticles seemed to depend on the temperature, pH, or kind of bacteria from which the supernatant was obtained, among other factors. Researchers observed that *A. kerguelensis* supernatant could not produce silver nanoparticles at the same temperature that *P. antarctica c*ould produce the same nanoparticles. This work provided significant evidence that the components in cell-free culture supernatants that encouraged the production of silver NPs varied from one bacterial species to another and that this was true across all bacterial species (Shivaji et al., 2011).

It is possible to synthesize AgNPs using this method, in which bacteria break down Ag^+^ to its elemental form (Ag^0^) outside the cell. Several shapes and sizes of AgNPs may be found in extracellularly produced AgNPs. These include hexagonal, spherical, triangular, circular, and cuboidal, depending on the culture medium utilised for the growth of bacteria (Nanda and Saravanan, 2009; Elbeshehy et al., 2015; Otari et al., 2015). The reducing agent for the biogenic reduction of Ag^+^ to Ag^0^ is the proteins on the bacterial cell wall or tiny soluble secretory enzymes. Extracellular synthesis of AgNPs by many bacterial taxa has been functionally described in the natural environment (Islam et al., 2021). *Aeromonas* sp. SH10 dry cell mass decreases Ag^+^ to Ag^0^ in the medium (Fu et al., 2006). Outside the cell, *Bacillus licheniformis, Bacillus pumilus,* and *Bacillus persicus* create AgNPs with a size range of 72–92 nm (Elbeshehy et al., 2015). Extracellular synthesis makes use of high-speed centrifugation (10,000 to 12,000 rpm) to capture AgNPs in solution, which may then be re-suspended in a variety of solvents once they have been recovered. Because of this, they are widely used in various fields, including optoelectronics, electrical circuits and systems, bioimaging, and sensory technologies.

When AgNPs are synthesized inside bacteria, silver ions are transported by membrane proteins. As a result of reducing Ag + to Ag^0^, certain silver-resistant bacteria limit their toxicity by accumulating Ag^0^ in the cell wall or periplasmic space (Murugan et al., 2014). Some studies have discovered up to 25 percent of the mass of Ag^0^ in the bacterial cell wall. *Pseudomonas stutzeri AG259* lowers the AgNO_3_ solution and produces AgNPs with a 200 nm size and a modest amount of monoclinic crystallised acanthite (Ag2S) (Murugan et al., 2014). AgNPs (varying in size from 10 to 15 nm) produced by *Corynebacteria* sp. *SH09* also forms a diamine Ag complex on the cell wall (Zhang et al., 2005). AgNPs created within cells must be recovered by additional stages such as bacterial cell lysis *via* ultrasonication, heat or chemical methods such as salt and detergents (Fesharaki et al., 2010; Kalishwaralal et al., 2010). Extracellular and intracellular synthesis of AgNPs by *Proteus mirablis* and *Vibrio alginolyticus*, respectively, has been observed in different media and growth conditions (Samadi et al., 2009; Rajeshkumar and Malarkodi, 2014). A study by Pugazhenthiran et al. [52] found that *Bacillus* sp. synthesised AgNPs (5–15 nm) in the medium and periplasm (Pugazhenthiran et al., 2009).

Several reports of Shewanella species are Gram-negative, polar flagellated, rod-like, and found in aquatic or marine environments (Bowman et al., 1997; Venkateswaran et al., 1999). Most *Shewanella species* are mesophilic, psychrotolerant, and psychrophilic bacteria (Zhao et al., 2005). *Shewanella alga* is a Gram-negative *bacillus* that may be found in water and soil, where it thrives (Kim et al., 2006). *S. algae* was used by Konishi et al. (Konishi et al., 2007) to deposit gold nanoparticles. In the presence of lactate or H_2_ as an electron donor and ferric citrate (III) citrate as an electron acceptor, *S. algae* bacteria can grow anaerobically. When H_2_ gas was used as an electron source, they showed that *S. algae* resting cells could reduce ions (1 mM) into elemental gold in 30 min at 25°C throughout a pH range of 2.0–7.0 (Caccavo et al., 1992). It was found that the periplasmic area of *S. algae* cells was filled with biogenic gold nanoparticles (10–20 nm). Extracellular gold NPs were deposited when the pH of the solution fell to <2.8. There was a wide range of biogenic gold NPs (15–200 nm) on the bacterial cells at this pH. Biogenic gold NPs (20 nm) were coated on the bacterial cells, and bigger gold particles (350 nm) were deposited extracellularly in a solution with pH 2.0. So it may be inferred that pH significantly impacts the form and location of biogenic gold nanoparticles (Konishi et al., 2007). The quick degradation of soluble gold [Au(III)] into insoluble gold was shown to be the likely cause of the drop in soluble gold concentration. Lacking H_2_ gas, S. algae cells failed to use lactate as an alternate electron source to decrease Au (III). Furthermore, H_2_ gas did not chemically reduce Au (III) in a sterile control condition devoid of *S. algal* cells. Thus, in the presence of molecular H2 as an electron donor, *S. algae* resting cells converted soluble Au (III) into insoluble gold (Kashefi et al., 2001; Konishi et al., 2006).

The NADH-dependent reductase enzyme, which gives an electron and oxidizes to NAD, was used to produce Ag nanoparticles using *Pseudomonas stutzeri* AG259 bacteria. Biological reduction of Ag ions to nanoparticles occurs due to electron transfer (Ahmad et al., 2003). The extracellular manufacture of Au nanoparticles was achieved in Husseiny et al., who reduced Au ions using *Pseudomonas aeruginosa* (Husseiny et al., 2007). Other studies have also shown the lack of participation of biological enzymes. When *Bacillus megaterium* cells were dried, Liu and others synthesized Au nanoparticles (Liu et al., 2009). Sneha et al. conducted a similar investigation employing a *Corynebacterium sp*. and found that a nonenzymatic reduction mechanism was responsible for the creation of nanoparticles (Sneha et al., 2010). Many variables are thought to be involved in the decrease of nanoparticles.

For the first component, the cell wall contains organic functional groups that promote reduction, and for the second, the proper environmental conditions are required, including a suitable acidity and temperature (Lin et al., 2001). *Lactobacillus* sp. *A09* and *Bacillus megaterium D01*, for example, may convert Ag ions to silver nanoparticles through the interaction of functional groups on the cell wall (Lin et al., 2005). The pH and temperature considerably impact nanoparticle size, shape, and content (Hulkoti and Taranath, 2014). Smaller particle sizes, for example, have more distinct physicochemical features than larger ones.

Consequently, it is necessary to tune the synthesis parameters throughout the nanoparticle production process to improve the overall particle qualities. Because these two characteristics are critical to nanoparticle production, choosing the suitable culture medium for certain bacteria and the right metallic salt is essential (Roh et al., 2001; Nair and Pradeep, 2002). It has been proven that the size and shape of particles may be affected by the concentration of metallic salts and the pH of the medium. Au nanoparticles ranging in size from 10 to 20 nm were formed at low concentrations of AuCl_4_ at pH 6. The salt content was increased to create Au nanowires at pH 6 (He et al., 2008). Changing the pH to four also produced spheres and triangular nanometer-scale plates when the salt concentrations were diluted. There is a definite correlation between pH and nanoparticle shape during production (Husseiny et al., 2007). Other researchers also synthesized metallic nanoparticles using bacterial species are enlisted in Table 3.

**TABLE 3:** Literature overview of various nanoparticles synthesized using bacterial species.

Species	NPs	Size (nm)	Ref
*Bacillus brevis*	*Silver*	41–68	Saravanan et al. (2018a)
*Lactobacillus fermentum*		5–80	Sintubin et al. (2009)
*Pseudomonas* sp.		20–70	John et al. (2020)
*Chitinophaga chungangae*		—	Huq and Akter, (2021c)
*Sphingobium* sp. *MAH-11*		7–22 nm	Akter and Huq, (2020)
*Terrabacter humi* sp. *nov*		6–24 nm	Akter et al. (2020)
*Lysinibacillus xylanilyticus MAHUQ-40*		8–30 nm	Huq, (2020)
*Massilia* sp. *MAHUQ-52*		15–55 nm	Huq and Akter, (2021b)
*Arthrobacter bangladeshi* sp. *nov*		12–50 nm	Amdadul Huq and Akter, (2021)
*Paenarthrobacter nicotinovorans MAHUQ-43*		13–27 nm	Huq and Akter, (2021a)
*Streptomyces griseoplanus*		19–20	Vijayabharathi et al. (2018)
*Escherichia coli DH5α*	*Gold*	25	Du et al. (2007)
*Pseudomonas aeruginosa*		15–30	Husseiny et al. (2007)
*Rhodopseudomonas capsulata*		10–20	He et al. (2008)
*Streptomyces* sp.		90 (average)	Vinay Gopal et al. (2013)
*Kocuria flava*	*Copper*	5–30	Kaur et al. (2015)
*Shewanella oneidensis*		20–40	Kimber et al. (2018)
*Streptomyces* sp.		78–80 (average)	Hassan et al. (2019)
*Bacillus amyloliquefaciens*	*Titanium*	22.11–97.28	Khan and Fulekar, (2016)
*Bacillus megaterium*	*Zinc*	45–95	Saravanan et al. (2018b)

### Algae Assisted Synthesis

*Pterocladia capillacae, Jania rubins, Ulva faciata, and Colpmenia sinus* (El-Rafie et al., 2013) algae specie’s have been used to synthesize silver nanoparticles. The NPs were 7–20 nm in diameter and spherical in shape. Researchers believe their antibacterial action is caused by a blockage of bacterial cell processes caused by their adhesion to the cell wall. Recent studies have shown the antimicrobial efficacy of *Sargassum longifolium* alga-derived silver nanoparticles (Rajeshkumar et al., 2014). After 1 h of mixing AgNO_3_ and aqueous algal extract, the reaction mixture becomes brown. The nanoparticles showed an absorption peak at 440 nm for polydispersed silver nanoparticles. The pH of the reaction mixture has been shown to significantly influence the production of silver nanoparticles. At a lower pH (6.2), the reaction mixture changed color more slowly. With the rise in pH, the reduction process became more visible. With increasing concentrations of silver nanoparticles, the antifungal activity against *Aspergillus fumigatus, Candida albicans,* and *Fusarium* sp. was observed to rise.

*Pithophora oedogonia,* a fresh water green alga, has been used to synthesize silver nanoparticles diameter 25–44 nm. Carbohydrates, saponins, steroids, and proteins were shown to decrease AgNO_3_ to silver nanoparticles by IR spectroscopy and quantitative analysis of the extract. Compared with Gram-positive bacteria, they were shown to be more efficacious (Sinha et al., 2015).

The production of silver nanoparticles from the marine alga *Caulerpa racemosa* and their antibacterial efficacy against human diseases have also been reported by Kathiraven et al. (Kathiraven et al., 2015). *Staphylococcus aureus* and *Proteus mirabilis* bacteria were killed at a low 5–15 L (5–25 nm) silver nanoparticles having face-centered cubic shape. Using a combination of 14 bacteria and microalgae, silver nanoparticles were created. Even in the dark, extracellular polysaccharides were producing nanoparticles. Silver nanoparticles of varying sizes and morphologies were found, ranging from species to species (Patel et al., 2015). Six harmful microorganisms were used to assess the antibacterial activity. The cell membrane is damaged due to the production of free radicals.

Living cells of the *Euglena gracilis* microalga to produce gold nanoparticles by Dahoumane and others (Dahoumane et al., 2016). Like other marine algae, the biomaterial in the alga acts as a reducing agent, capping agent, and catalyst. The yield of nanoparticles is influenced by several variables, including pH, reaction time, temperature, and concentration. Gold nanoparticle generation and release are thought to proceed in three steps: 1) absorption of Au^+3^; 2) reduction of Au^+3^ to Au^0^; and 3) release of gold nanoparticles into the solvent. This theory has been widely accepted. As a result of their distributed nature, they don’t collect in one place. Their dimensions range from 10 nm to several hundred nanometers. This shows that all algae have a tolerance limit and a certain ability to decrease metal ions to protect them from the poisonous impact of Au^3+^/Au^0^, since an AuCl_3_ concentration of 10^3^ M is deadly to *E. gracilis.*

*Stoechospermum marginatum* biomass has been shown to produce gold nanoparticles biogenically (Arockiya Aarthi Rajathi et al., 2012). Within 10 min of adding HAuCl_4_, the brown extract became ruby red, displaying an absorption at 550 nm in the UV-vis spectrum due to SPR (Singaravelu et al., 2007). These polydispersed nanoparticles were cylindrical primarily, hexagonal, and triangular, with a diameter of 18.7–93.7 nm in the TEM pictures. On the other hand, SEM scans revealed the development of 40–85 nm gold nanoparticles. Algal extracts include terpenoids and phenols, which function as catalysts to convert gold ions into gold nanoparticles. A face-centered cubic gold structure was discovered using X-ray diffraction (Shankar et al., 2003).

In recent years, a limited number of researchers have reported the manufacture of Ag nanoparticles utilizing numerous kinds of seaweed. There have been reports of the biosynthesis of Ag nanoparticles using *Padina tetrastromatica* (brown seaweed), for example (Rajeshkumar et al., 2012). According to their research, the nanoparticles were spherical in form, with a mean particle size of 14 nm, and showed antibacterial properties. From *Codium capitatum*, the crystalline Ag nanoparticles were also biosynthesized in the range of 3–44 nm, with an average particle size of 30 nm (Kannan et al., 2013). *Spirogyra insignis*, another green alga, created spherical nanoparticles with a mean particle size of 30 nm (Castro et al., 2013), whereas *Padina tetrastromatica*, a macroalga, produced crystalline spherical nanoparticles with a size range of 5–35 nm (Gopinath, 2015). Although the antifungal, antibacterial, and anticancer effects of Ag nanoparticles generated by seaweeds have also been discovered. Biosynthesis of both Ag and Au nanoparticles has been achieved by Ramkumar Vijayan et al. using an aqueous solution containing an extract from *Turbinaria conoides*. The nanoparticles were also tested for their ability to inhibit the development of biofilms (Nag et al., 2021). Antimicrobial nanoparticles derived from a *Sargassum plagiophyllum* aqueous extract have also been demonstrated to be effective against bacterial pathogens such as *Escherichia coli* (Stalin Dhas et al., 2014).

Romero-González et al. found that de-alginate seaweed debris may be employed as a catalyst for reducing Au ions in solution to generate Au particles ranging in size from the nanometers to roughly 6 μm. It was discovered that the functions found in seaweed were effective in generating stable particles with a wide range of forms such as hexagonal and decahedral plates and rods, as well as irregular and decahedral rods (Romero-González et al., 2003). An example of an eco-friendly approach to extract Au from hydrometallurgical solutions was shown by Mata et al. in a similar investigation. Brown seaweed (*Fucus vesiculosus*) was used to biosorb and bioreduce Au, resulting in nanoparticles of varied sizes and morphologies (Mata et al., 2009). *Sargassum wightii Greville*, a marine alga, has also been used to produce Au nanoparticles by Singaravelu et al. (Singaravelu et al., 2007). Alga generated stable nanoparticles that ranged from 8 to 15 nm in diameter and were spherical. Researchers have been able to produce a wide range of stable nanoparticle sizes in similar studies by Luangpipat et al. using *Chlorella vulgaris* (Luangpipat et al., 2011), Rajasulochana et al. (*Kappaphycus alvarezii*) (Rajasulochana et al., 2010), and Stalin Dhas et al. (*Sargassum myriocystum*) (Stalin Dhas et al., 2012), while Senapati et al. have reported the biosynthesis of Au nanoparticles (Senapati et al., 2012). Green alga *Spirogyra insignis* and red alga *Chondrus crispus* were used by Castro et al. to synthesize Au nanoparticles (Castro et al., 2013). A brown alga, *Stoechospermum marginatum*, was used to biosynthesize gold nanoparticles by *Arockiya Aarthi* Rajathi et al. Their analysis found that the nanoparticles were crystallized and varied in size from 18.7 to 93.7 nm, with a limited number of hexagonal and triangular platelets in the mix. Diterpenoids in brown seaweed were discovered to be directly engaged in reducing Au by the hydroxyl groups. The nanoparticles also showed antibiotic efficacy against various bacterial pathogens (Arockiya Aarthi Rajathi et al., 2012). For example, brown seaweeds (*Turbinaria ornate* and *Padina pavonica*) exhibit biosynthesized Au nanoparticles ranging in size from 7 to 11 nm (Ashokkumar and Vijayaraghavan, 2016) and from 30 to 70 nm (*Padina pavonica*). The biosynthesis of gold nanoparticles by two freshwater algae species has also been shown (Sharma et al., 2014a; 2014b). These species are the green alga *Prasiola crispa* and the red alga *Lemanea fluviatilis*.

Abboud et al. reported a biosynthesis of copper oxide nanoparticles utilizing a brown alga extract (*Bifurcaria bifurcata*). Nanoparticles of cuprous oxide (Cu_2_O) and cupric oxide (CuO) were generated in a simple technique. A few nanoparticles were elongated, but the vast majority of the particles were spherical. An average particle size of 22.6 nm was discovered for the samples, which varied from 5 to 45 nm. Copper oxide nanoparticles were shown to be effective against both *Enterobacter aerogenes* and *Staphylococcus aureus* in subsequent antibacterial experiments (Abboud et al., 2014). The manufacture of copper cored copper oxide nanoparticles utilizing red seaweed extracts (*Kappaphycus alvarezii*) was also described in a recent work by Khanehzaei et al. Stabilized copper cored-cuprous oxide nanoparticles with a mean particle size of 53 nm were synthesized in the presence of seaweed. Nanoparticle surfaces were also discovered to be capped by pairs of electrons, some hydroxide and sulphur groups from the water-soluble sulphated polysaccharides present in seaweed cell walls (Khanehzaei et al., 2015).

One-step green biogenic synthesis of ferric oxide (Fe_3_O_4_) nanoparticles using brown seaweed was recently shown in work by Mahdavi and others (*Sargassum muticum*). To make Fe_3_O_4_ nanoparticles, an aqueous seaweed extract was combined with an aqueous ferric chloride solution. Reduction and capping are both accomplished by the amino, carboxy, and hydroxyl functional groups produced from the water-soluble polysaccharide cell walls (Mahdavi et al., 2013). The average size of the particles formed was 18 nm, crystalline, and cubic in shape. It was shown that the Fe_3_O_4_ nanoparticles generated by Namvar et al. have anticancer efficacy against human cancer cell lines, including leukemia, breast cancer, cervical cancer, and liver cancer, when used *in vitro* tests. The buildup of Fe_3_O_4_ nanoparticles in treated cells was shown to increase cell death *in vitro* tests and proved their potential utility in cancer therapy (Namvar et al., 2014).

Another work used the marine green alga *Caulerpa serrulate* to bio fabricate stable colloidal crystalline AgNPs. The manufactured NPs were found to be between 10 and 2 nm in diameter and spherical in form by TEM. The photocatalytic activity was shown, with 99 percent of Congo red dye degraded after only 6 min of incubation. They also showed antibacterial action against Gram-negative and Gram-positive bacteria, including *Staphylococcus aureus, Shigella* sp.*, Salmonella typhi,* and *Escherichia coli* (Aboelfetoh et al., 2017). Another complex dye, methylene blue (MB), is hazardous to living beings, making its breakdown a critical concern for both the environment and biology. After 30 min of exposure to light and NaBH_4_, Edison et al. could bio generate AgNPs that could totally break down MB in the presence of the marine green alga *Caulerpa racemosa* (Edison et al., 2016).

Bioactive molecules for the production of AgNPs have been discovered by combining a sulfated polysaccharide obtained from the marine red alga *Porphyra vietnamensis* with silver nitrate. Antibacterial activity against Gram-negative and Gram-positive bacteria was shown by the production of NPs with an average diameter of 13 nm (Venkatpurwar and Pokharkar, 2011).

A chloroauric acid (HAuCl_4_) solution and an aqueous extract of marine microalgae (*Tetraselmis suecica*) were used to synthesize and characterize gold nanoparticles (AuNPs). There was a distinct band in the UV–Vis spectrum that corresponded to the formation of AuNPs. However, the most common 79 nm diameter with a polydispersed and crystalline structure (Shakibaie et al., 2010). Their diameter ranged from 51 to 120 nm. Polysaccharide hydroxyl groups from the algal polysaccharides were shown to have an essential role in the biosynthesis of AuNPs from *Padina gymnospora.* The generated NPs’ crystalline nature was verified by X-ray diffraction (XRD), and an AFM study showed that they were between 53 and 67 nm in size (Singh et al., 2013).

Ramakrishna and his colleagues used Sargassum tenerrimum and Turbinaria conoides as reducing agents for gold ions. Two extracts of the gold nanoparticles showed photocatalytic activity by degrading 4-nitrophenol and p-nitroaniline into their corresponding aminoarenes (4-aminophenol and p-phenylenediamine) and rendering naturally coloured solutions (Rhodamine B and Sulforhodamine) into colorless solution in the presence of NaBH_4_ as a catalyst (Ramakrishna et al., 2016). Because of the additive action of nanosilver and the wide range of phytoconstituents with intrinsic antimicrobial capabilities, silver nanoparticles with exceptional stability and environmental friendliness can be easily manufactured from plant extracts and demonstrate a broad spectrum of antimicrobial activities, anticancer activities and catalytic reduction of 4-nitrophenol (Bharadwaj et al., 2021). Nanoparticles synthesized using algae are enlisted in Table 4.

**TABLE 4:** Tabular representation of nanoparticles synthesized using algae.

Algae	NPs	Size (nm)	References
*Acanthophora spicifera*	Au	<20	Ramakrishna et al. (2016)
*Caulerpa racemosa*	Ag	25	Edison et al. (2016)
*Caulerpa serrulata*	Ag	10 ± 2	Aboelfetoh et al. (2017)
*Chaetomorpha* sp.	Ag	15	Karimi and Samimi, (2019)
*Chaetomorpha antennina*	Fe	10–18	Sreelakshmi et al. (2020)
*Codium tomentosuml*	Ag	20–40	Murugan et al. (2016)
*Corallina elongate*	Ag	12–20	(Hamouda et al., 2019)
*Dictyota dichotoma*	Ru	25–90	Ali et al. (2017)
*Ecklonia cava*	Au	20–50	Venkatesan et al. (2014)
*Enteromorpha intestinalis*	Ag	10–20	Raju et al. (2017)
*Fucus vesiculosus*	Au	10–100	Vijayakumar et al. (2017)
	Au	73–96	Manivasagan et al. (2016)
*Gracilaria verrucose*	Au	20–80	Chellapandian et al. (2019)
*Gelidium amansii*	Ag	8–25	Hamouda et al. (2019)
*Gelidiella acerosa*	Au	5.81–117.59	Senthilkumar et al. (2019)
*Gelidium amansii*	Ag	27–54	Pugazhendhi et al. (2018)
*Gelidium corneum*	Ag	20–50	Yılmaz Öztürk et al. (2020)
*Gelidium pusillum*	Au	7–17	Jeyarani et al. (2020)
*Hypnea musciformis*	Ag	40–65	Roni et al. (2015)
*Laurencia papillosa*	Ag	NR	Omar et al. (2017)
*Sargassum coreanum*	Ag	19	Somasundaram et al. (2021)
*Chaetomorpha linum*	Ag	NR	Acharya et al. (2020)
*Oscillatoria princeps*	Ag	NR	Bishoyi et al. (2020)
*Nannochloropsis oculate*	Ag	11.6–26.1	El-Kassas and Ghobrial, (2017)
Red algae	Au	35 ± 8	Chen et al. (2018)
Red algae	Co_3_O_4_	>30	Ajarem et al. (2021)
*S. myriocystum*	ZnO	76–186	Nagarajan and Arumugam Kuppusamy, (2013)
*S. wightii*	ZrO_2_	4.8–5	Kumaresan et al. (2018)
	ZnO	40–50	Ishwarya et al. (2018)
	Ag	8–27	Govindaraju et al. (2009)
*Trichodesmium erythraeum*	Ag	26.5	Sathishkumar et al. (2019)
*Turbinaria conoides*/Aqueous	Au	27.5 12–57	Ramakrishna et al. (2016)
	Au	60	Rajeshkumar et al. (2013)
*U. lactuca*/ethyl acetate	Ag	3–50	Sahayaraj et al. (2019)

## Factors Affecting Biosynthesis of Nanoparticles

There are a number of elements that influence the formation and shape of nanoparticles that have been developed. Researchers have linked these variances to the synthetic process’s choice of adsorbate and catalyst (Patra and Baek, 2014). Nanoparticles creation from biological extracts may also be affected by reaction conditions. Studies have shown that a reaction solution’s pH has a significant impact on the production of the nanoparticles that result. The form and size of the generated nanoparticles may be affected by changes in the reaction pH. When comparing lower acidic pH values to higher acidic pH values, bigger particles are produced. The bigger particles (25–85 nm) were generated at pH two whereas the smaller particles (5–20 nm) were created at pH three and four in a research using *Avena sativa* biomass (Armendariz et al., 2004). Particle aggregation may have been caused by the lack of functional groups at pH 2, according to the researchers. The bacteria *Rhodopseudomonas capsulate* was shown to produce gold nanoparticles in a similar manner. It was discovered that, with a pH rise of 7, spherical particles measuring 10–20 nm were present. Nanoplates were formed when the reaction pH was lowered to 4 (He et al., 2007).

Another researcher demonstrated that the pH of Saudi Dates extract had an impact on the shape, reaction rate, and size of biosynthesized Pt NPs(Al-Radadi, 2019). The reaction rate was found to be quicker when the dispersive medium’s hydroxyl content rose. The acidified media, on the other hand, created a variety of different-sized particles. Shape and size of synthesised Pt NPs are expected to be rod-shaped at pHs 1.5, 3.5, 5, and 7, with a diameter of 700.5 nm, spherical at sizes 5.0–5.4 nm, 2.5–13.8 nm, and rod-shaped at pHs 1.5–5.5 with 700.5 nm diameter.

Another key part of any synthesis is temperature. Temperature increase has showed catalytic behaviour by boosting the reaction rate and efficiency of nanoparticle synthesis while using biological entities to formulate nanoparticles. According to a research on neem leaf extracts and the production of AgNPs, temperature elevation (10–50 °C) was linked to an increase in the reduction of Ag+(Verma and Mehata, 2016). Smaller AgNPs were formed at 50°C in the same way as Kaviya et al. found in the generation of AgNPs from citrus peel extract using different temperatures (Kaviya et al., 2011). AgNPs were also produced in this manner from *Escherichia coli* wasted culture supernatants (Gurunathan et al., 2009). The scientists speculated that a critical enzyme involved in the creation of nanoparticles may have been affected by elevated temperatures. But the study’s findings showed that temperatures over 60°C favoured the creation of larger-sized particles, which was surprising. Molecular kinetics at high temperatures causes fast reduction of Ag+ (which aids reduction and nucleation) at the expense of secondary reduction on nascent particle surfaces, which is why this finding was made. At higher incubation temperatures. Saudi’s dates extract was used by Al-Radadi to study the effect of temperature on the biogenesis of Pt NPs. The average particle size was 3.4 nm at 20°C and 2.6 nm at 30°C, according to microscopy measurements (Al-Radadi, 2019). It has also been shown that temperature has an effect on the structure of nanoparticles as well. While AgNPs were generated at ambient temperature using *Cassia* fistula extracts, spherical AgNPs were created at higher temperatures (over 60 °C) (Lin et al., 2010). Plant macromolecules’ interactions with Ag faces were assumed to be altered by high temperatures in the research, which prevented the coalescence of nearby nanoparticles.

## Applications of Biosynthesized Metallic Nanoparticles

### Silver Nanoparticles

Silver has a long history of usage as an antibacterial, and its nanoforms are significantly superior and more biocompatible antimicrobial agents now than their conventional counterparts. Research on silver consumption in nanotechnology has been going on since the dawn of the age of nanotechnology, and this section covers a lot of that work. Silver nanoparticles have received a great deal of attention in recent years for their potential to combat infectious diseases by closing the gaps in current antimicrobial formulation techniques, eradicating drug-resistant microorganisms, and establishing a foothold for the emerging field of conjugated silver nanoparticles. This research could help us better understand the role of silver nanoparticles in future antimicrobial treatments. Because of the additive action of nanosilver and the large variety of phytoconstituents with intrinsic antimicrobial capabilities, silver nanoparticles may be readily manufactured from plant extracts with extraordinary stability and environmental friendliness and demonstrate a broad spectrum of antimicrobial activities.

In research by Loo et al., silver nanoparticles (4.06 nm) produced from Puerh tea leaves have been discovered to have high antibacterial efficacy against Gram-ve pathogenic pathogens such as *Klebsiella pneumonia, Escherichia coli*, and *Salmonella typhimurium.* Furthermore, the Minimum inhibitory concentrations (MICs) recorded were: 3.9, 3.9, 7.8, and 3.90 μg/ml (Loo et al., 2018). Silver nanoparticles (quasi-spherical and spherical; 5 nm in size) were synthesized by Garibo et al. using an aqueous extract of the perennial tree *Lysiloma acapulcensis* and were proven to be powerful antibacterial agents in their research (Garibo et al., 2020; Rahman et al., 2021a). Selim et al. studied the antimicrobial activity of silver nanoparticles with an average size of 50 nm on *M. tuberculosis*, MDR *Mycobacterium tuberculosis* strain, and clinical isolates in important research (*M. tuberculosis* and *M. bovis*). All of the investigated substances were strongly inhibited by the produced nanoparticles. *M. tuberculosis* MIC values were determined to be 4 μg/ml and 1 μg/ml for *M. bovis* and *M. tuberculosis* correspondingly. *M. tuberculosis* MDR strain has a MIC value of 16 μg per litre. However, MIC values of 1–16 μg/ml and 4–32 μg/ml have been recorded for *M. tuberculosis* and *M. bovis* clinical isolates, respectively. According to the findings of this investigation, silver nanoformulations may have the antitubercular potential (Selim et al., 2018). Using synthesized silver nanoparticles, Singh et al. found that antibacterial activity of bacteriogenic silver nanoparticles against the pathogenic nosocomial *Acinetobacter baumannii* was inhibited with a MIC value of 16 μg/ml, significantly lower than the MIC values of ampicillin (4,096 μg/ml), amoxicillin (2048 μg/ml), and erythromycin (64 μg/ml). This research reveals that AgNPs have more promise and effectiveness in treating hospital-acquired illnesses than traditional antibiotics (Singh et al., 2017).

Proton motive force strength generated by the ionic connection between AgNPs and the bacterial cell wall may interrupt the activity of enzymes containing thiol groups (Sereemaspun et al., 2008). When used against *E. coli,* Avicennia marina-based AgNPs were shown to have bactericidal effects because the proton motive force may have been dissipated (Gnanadesigan et al., 2012). The effect of AgNPs on the cell membrane and cell is dependent on the cell’s makeup. Using *Argemone mexicana* leaf extract and antibacterial experiments with *E. coli* and *P. aeruginosa*, Singh et al. synthesized green nanoparticles in their work (Singh et al., 2010). According to the research, both the bactericidal effects of the AgNPs and the polymer subunit’s potential to break membranes were shown to have antibacterial properties.

In comparison to Gram-negative bacteria, Gram-positive bacteria have a stronger cell wall due to a lower concentration of lipopolysaccharides, making them a more formidable barrier to the entry of AgNPs. Gram-negative bacteria’s cell walls and membranes are thinner due to more lipopolysaccharides and less peptidoglycan. They adhere to AgNPs due to their composition, stability, and negative charge. Because AgNPs have an electrical affinity to bacteria, they may be used to kill them, as was previously stated (Abbaszadegan et al., 2015). A change in the cell’s internal environment and membrane polarization is caused by this attraction and activity, resulting in cell death (Abalkhil et al., 2017). In contrast to bacteria that aren’t exposed to AgNPs, those that display morphological and physiological alterations, and their integrity is disrupted. The components of the bacterial cell, such as nucleic acid, proteins, enzymes, metabolites, and the bacterial cell’s energy supply, are released into the environment when the cell wall and membrane are ruptured (Gomaa, 2017).

AgNPs attach to proteins and DNA in bacteria and induce conformational changes, resulting in less stable states that limit their ability to function (Bondarenko et al., 2013). According to studies, DNA degradation is facilitated by sulphur and other amino groups on the membrane surface. The bacteria are affected due to the interaction between the AgNP and the bacterium-infected macromolecules (DNA, protein, and lipids). AgNPs derived from *Datura stramonium*, for example, were shown to exhibit antibacterial action against *E. coli* by Gomathi et al. (Gomathi et al., 2017) (2017). According to the findings of this research, Ag ions from AgNPs enter bacterium cells, where they cause significant damage and eventually lead to cell death. In a similar vein, AgNPs generated from *Urtica dioica* interacted with bacterium cells and penetrated them, releasing Ag^+^ ions that inhibited DNA replication and eventually killed the bacteria, as described by Jyoti et al. (Jyoti et al., 2016).

**FIGURE 2:**
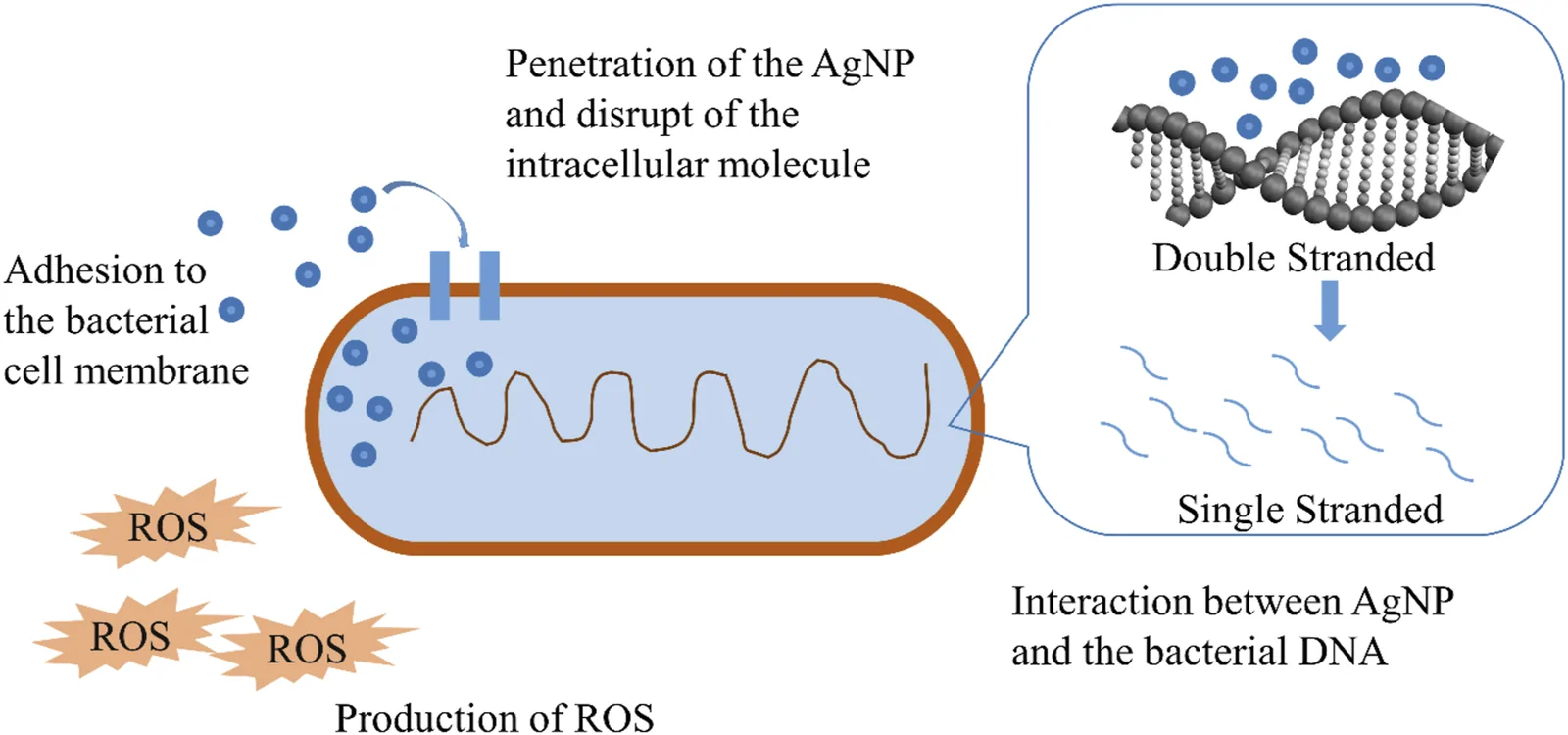
Antibacterial action of silver nanoparticles *via* ROS pathway.

Antimicrobials may cause oxidative stress in bacteria by introducing them to the cell. The generation of reactive oxidative species (ROS) in resistant bacteria is one method of inhibiting their proliferation. Antimicrobials are often used to raise ROS levels. However, the introduction of AgNPs to resistant microorganisms elevates ROS levels, leading to resistant species, according to a recent study. Based on the findings of Khan and Ali (Khan and Ali, 2020), it seems that the introduction of AgNPs to resistant microorganisms such as *Xanthomonas citri, S. aureus*, and *Erwinia carotovora* causes ROS levels to rise beyond critical levels. The study found that ROS levels rose when AgNPs were added to bacterial suspensions, resulting in bacterial suppression. AgNPs caused the rise in ROS when exposed to resistant strains of *E. coli* and *S. aureus*, according to Das et al. (2017). The research found that when these resistant strains are treated with AgNPs, the ROS levels in these bacteria rise, resulting in cellular inhibition (As shown in Figure 2). According to Kim et al. (2011), AgNPs generated ROS that damaged the cell membrane, protein, and DNA of *E. coli* and *S. aureus* (Kim et al., 2011).

Oyster mushroom AgNPs were shown to be superior to *S. aureus* in terms of antibacterial activity against *E. coli* and *Pseudomonas aeruginosa* (Nithya and Ragunathan, 2009). It was shown that *P. ostreatus*-AgNPs effectively against Gram-positive bacteria such as *S. aureus* when evaluated by disc diffusion technique (Mirunalini et al., 2012). It was found that the most significant inhibition zone of *P. ostreatus*-AgNPs was 8 mm. The inhibitory zone was smaller in oyster mushroom extracts than silver nanoparticles. Ag^+^'s antibacterial properties have not been fully elucidated; however, it may be due to the electrostatic attraction between nanoparticles’ positive and bacteria’s negative charges (Mirunalini et al., 2012). The antibacterial properties of AgNP made it a contender for use as an antimicrobial agent.

*Syzygium cumini*, also known as “Jammun” in Hindi, is a member of the plant family Myrtaceae and is sometimes referred to as “Indian blackberry.” *Syzygium cumini*, a common medicinal herb, is used to treat a broad range of ailments. Since ancient times, people have utilised the bark of this herb to cure a wide range of ailments, from sore throats and bronchitis to asthma and diarrhoea to ulcers and stomach ailments including diarrhea and biliousness. *Syzygium cumini* extract was used by Chakravarty et al. to synthesize silver nanoparticles with anti-inflammatory, antibacterial, and antioxidant properties (Chakravarty et al., 2022). DPPH free radical quenching characteristics of these green nanoparticles were found to be excellent. Anti-inflammatory silver nanoparticles that are green or bio-synthesized are likewise effective, with an 82.7 percent inhibition of albumin denaturation at a 1000-μg-per-liter concentration (Chakravarty et al., 2022).

Flowering plant and member of the Portulacaceae family *Portulaca oleracea L*. is often known as Purslane, the duckweed or small hogweed. Al-Otibi et al. created two types of green AgNPs, one by irradiating previously prepared AgNPs with ^60^Co *γ*-ray while utilising chitosan or by combining the aqueous extract of P. oleracea with silver nitrate (AgNO3) (normal AgNPs) (gamma-irradiated AgNPs) (Al-Otibi et al., 2022). *Curvularia spicifera, Macrophomina phaseolina*, and *Bipolaris* sp. were all shown to be plant pathogenic fungus. Compared to the regular AgNPs, the irradiation green AgNPs demonstrated a higher antifungal impact against all three of the tested fungal strains, with just a few outliers. There were noticeable changes in the fungal strains after exposure to the two AgNP formulations, including flaccid structures and compacted hyphae. Against *C. spicifera, M. phaseolina*, and *Bipolaris* species, the biosynthesized *P. oleracea* AgNPs seemed to exhibit antifungal activities. It is possible that these AgNPs might be used as a fungicide to protect a variety of plants against pathogens.

### Gold Nanoparticles

*C. aromaticus* leaf extracts were utilised to create the Au NPs, then employed as antibacterial agents. Temperatures (at 30, 60, and 100°C) were used to control the generation of NPs. It was shown that *C. aromaticus*-prepared Au NPs had strong antibacterial activity (ABA) when tested against various strains of bacteria (Boomi et al., 2019). For this investigation, nigra leaves from *A. nigra* were also used to make AuNPs, and their ABA content was tested. Bacteria (Gram-positive and Gram-negative) could not grow in the presence of Au nanoparticles, measured to be 21.52 nm in size. *A. nigra*, according to the findings, may be able to produce nanoparticles of gold (Au NPs) that serve as antibacterial agents (Baruah et al., 2018). Cashew leaves extract was also employed to synthesis antibacterial agents, and it was shown that the biogenically produced Au NPs were quite effective against both *B. subtilis* and *E. coli* bacteria (Sunderam et al., 2019). Gold nanoparticles produced from *M. koenigii* leaf extract showed a significant level of ABA activity.

Au NPs were synthesized using green and black tea leaf extracts, and their ABA activity was investigated. Room-temperature-prepared Au NPs had a diameter of 10 nm and were influential in treating bacterial strains (Onitsuka et al., 2019). Au NPs may also be generated at room temperature using *C. japonica* leaf extract. To see whether it could compete with regular antibiotics against bacteria like *K. pneumoniae* and *E. coli and the common cold and flu virus and Staphylococcus aureus and Candida albicans and Proteus mirabilis* the Au NPs’ ABA was put to the test (Sharma et al., 2019). The aqueous leaf extract of *G. superba* was used to fabricate Au NPs, and the NPs were spherical and 20 nm in size (Gopinath et al., 2016). *G. superba* aqueous leaf extract was proposed to synthesize Au NPs to develop medications efficient in treating microbial toxins based on data showing great ABA against bacteria (Gram-positive and negative) (Gopinath et al., 2016).

Au NPs were prepared at low temperatures using *P. atlantica* leaf extract, and the antibacterial properties of the NPs were also tested. Particles with a diameter of 50–60 nm were used. Diffusion technique was employed to assess the antibacterial activity of Au NPs, and the broth dilution method was used to determine the MIC and Minimum Bactericidal Concentration (MBC). The ABA of the Au NPs was encouraging, and the MIC value was also relatively low, equivalent to that of a conventional antibiotic (Hamelian et al., 2018). The aqueous leaf extract of *N. nouchali* was utilized to synthesize Au NPs, and the same NPs were used for the ABA assessment against *E. coli*, as previously reported. *N. nouchali* leaf extract extracts were used to manufacture 54.7 nm-sized AuNPs, which showed promise in therapeutic effectiveness (Maji et al., 2019). Au NPS synthesis was also carried out using *A. bettzickiana* leaf extract. *S. typhi, B. subtilis, P. aeroginosa, E. aerogenes, S. aureus,* and *M. luteus* were tested against the ABA particles ranging from 80 to 120 nm in diameter (Nagalingam et al., 2018). The ABA of Au NPs generated by fresh/dry leaf extract of *M. indica* extracts was also tested by Philip (Philip, 2010). These particles were produced using a cost-effective, repeatable green approach with 17–20 nm average diameter. A survey of bacterial species demonstrated that the produced Au NPs had promising ABA.

There has been significant interest in gold nanoparticles because of their unusual inertness to the external environment. Biosensing and medication delivery might benefit significantly from its properties (Elahi et al., 2018). It is also used to destroy potentially hazardous dyes to demonstrate its photocatalytic properties. Methyl Blue and Methyl Orange colors were reduced after 12 min of adding Au NP solution, according to Kumar et al., While Direct Blue 24 took just 18 min (Bogireddy et al., 2015). The breakdown of environmentally harmful colours like Methylene Blue has been made possible with certain plants. The paper, textile, and rubber sectors employ this basic cationic dye. The gold NPs mediated by the floral extract of Plumeria alba efficiently eliminated all of the Methylene blue dye. Degradation takes 40 min for a 5 percent solution and 70 min for a 1 percent solution to be complete (Mata et al., 2016).

It was found that plant-AuNPs could be easily synthesized utilizing the leaf extracts of *Paederia foetida* Linn (Bhuyan et al., 2017). When decomposing rhodamine B in an aqueous solution with NaBH4, the AuNPs showed photocatalytic efficiency under Sun irradiation. At a wavelength of 554 nm, UV-vis spectroscopy revealed the whole process. During the photocatalytic process, the dye solution lost its bright pink hue. It became colorless, showing that the AuNPs played an essential role in producing structural alterations in the rhodamine B dye and deleting chromophoric groups linked to dye molecules. Adsorption of BH_4_ on the surface of AuNPs induced electron transfer from BH_4_ to rhodamine B through AuNPs, which was the catalytic process.

AuNPs-based sensors were created by reducing HAuCl_4_ using *Solanum lycopersicums* juice extract as a reducing agent. The plant-AuNPs had an SPR peak at 546 nm, red-shifted to 800 nm, and lost strength when Cu^2+^ was added to the mixture, as seen in the UV-vis spectrum (Duan et al., 2019). Purple had become blue in the reaction, indicating the presence of AuNPs and Cu^2+^ ions interacting. An SPR peak that appeared at 800 nm resulted from molecules adsorbing onto AuNPs and causing them to clump together. Bonds produced between the AuNPs containing Cu^2+^ and the biomolecules in the juice extract from *Solanum lycopersicum* were used as linkers to bind the biomolecules created. These materials might detect Cu^2+^ ions in water purification by aggregating AuNPs and changing their color further.

*Smilax glabra* rhizome extracts preserved AuNPs, and the therapeutic impact of the AuNPs produced from *Smilax glabra* on obese and diabetic rats was studied (Ansari et al., 2019). Results from histopathological studies showed plant-AuNPs therapy repaired the nuclei and membranes of diabetes cells, as well as the cells’ cytoplasm. As well as, AuNPs stabilized with extracts of *Vetex negundo* and *Camellia sinensis* leaves were utilised to treat acute myeloid leukemia in mice models and to test the efficacy of pro-apoptotic agents on human gastric cancer cells.

### Zinc-Based Nanoparticles

**FIGURE 3:**
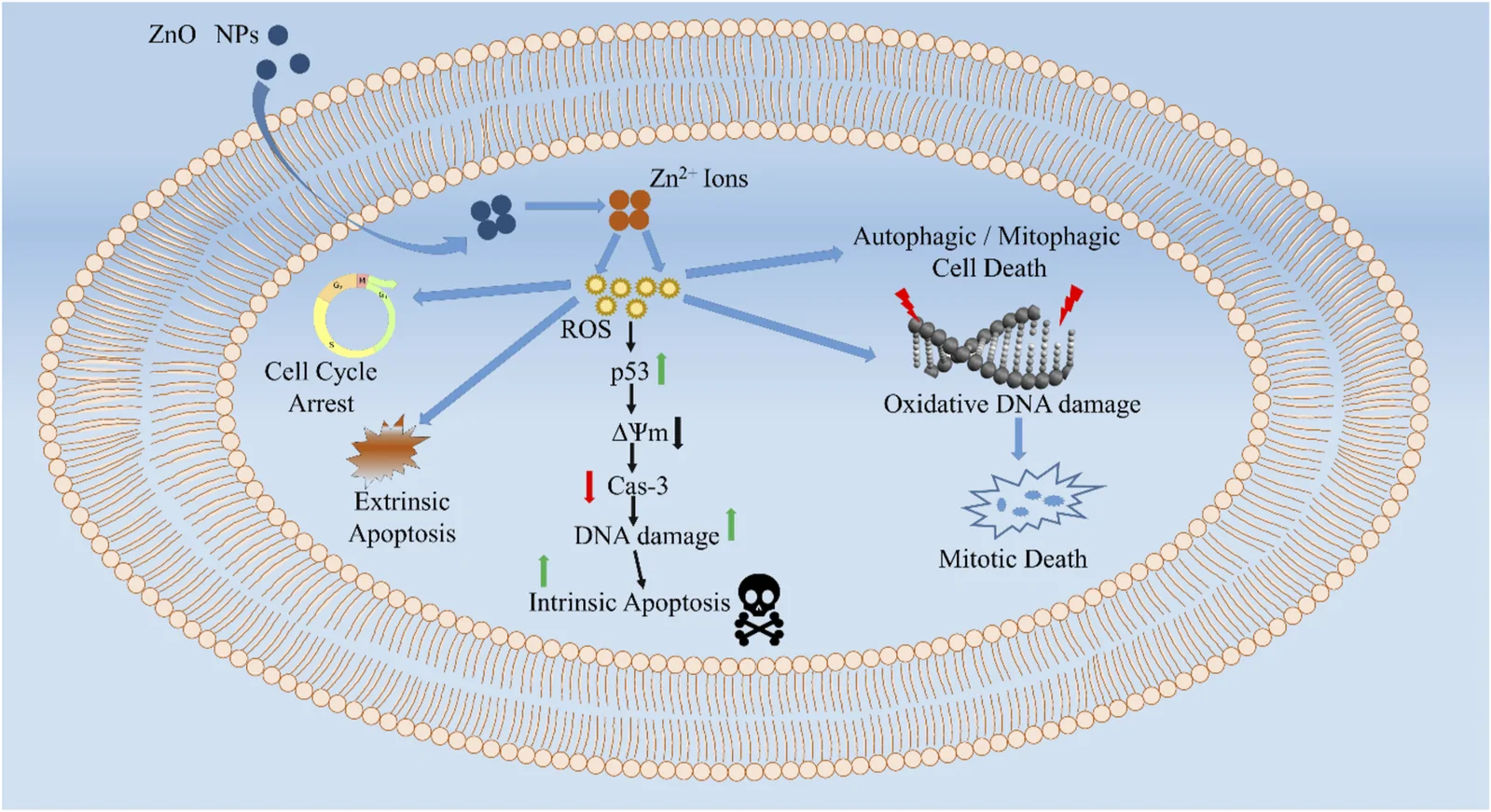
Anticancer effect of zinc nanoparticles.

To study the cytotoxic impact of green ZnO NPs on MG-63 osteosarcoma cancer cells, the cells were exposed for 24 h at doses of 1–100 μg/ml (Sisubalan et al., 2018). A concentration of 10 μg/ml of ZnO NPs is sufficient to kill 50% of the cells. The release of reactive oxygen species (ROS) by ZnO NPs causes morphological damage and cell death. Non-melanoma skin cancer cells (A431) and normal Vero cells were tested for cytotoxicity of green produced ZnO spherical and sheet-like NPs, respectively (Jevapatarakul et al., 2020). Dose-dependent cytotoxicity of ZnO (30–150 g/ml) was seen in lines A431 at the highest dosage, resulting in cell death of 40–50% in 48 h without harming normal Vero cells. Targeted medication delivery using ZnO nanoparticles provides new options for cancer therapy that are both safer and more effective. Zinc oxide nanoparticles (ZnO) may be used as nanocarriers for various chemotherapeutic drugs that synergistically impact cancer cells (As shown in Figure 3). A new nanocomposite of Cur/PMMA-PEG/ZnO NPs was developed by Dhivya et al. to transport curcumin and improve its solubility and cytotoxicity. PMMA-PEG/ZnO nanocomposite with an average size of less than 80 nm was shown to release curcumin faster under acidic pH 5.8. The IC50 for human gastric cancer AGS cells was 0.01 g/ml for the Cur/PMMA-PEG/ZnO nanocomposite (Dhivya et al., 2017).

### Anticancer Effect of Zinc

Nanoparticles with antifungal activities have been extensively researched and published. Among them, phytogenic ZnO NPs are the most widely studied because of their biocompatibility and flexibility. The antifungal agent ZnO NPs was produced utilizing surfactant extracted from seeds of *Acacia concinna* (Sharma et al., 2010). An aloe-broth-mediated ZnO NP was evaluated against various aspergilli such as *Aspergillus flavus, Nidulans, Trichoderma harzianum*, and *Stolonifer.* Fungicidal efficacy against the tested fungus strains was shown using ZnO NP suspensions with concentrations between 8 and 20 mM (Gunalan et al., 2012). To combat a wide range of fungus species found in a drinking water pipeline, ZnO NPs were synthesized using the stem extract of *Boswellia ovalifoliolata. Meyerozyma caribbica, Aspergillus parvisclerotigenus, Meyerozyma guilliermondii, Rhizopus oryzae, Aspergillus oryzae*, and *Trichoderma asperellum* were all successfully treated with ZnNPs (Supraja et al., 2016). *L. aculeata* extract was used to synthesize ZnO NPs that were well disseminated and showed potential antifungal efficacy against *A. niger, F. oxysporum*, and *P. funiculosum* (Narendhran and Sivaraj, 2016). To make biofuel-grade ZnO NPs, *Moringa oleifera* extract was used in the extraction process (Surendra et al., 2016). On *Alternaria saloni* and *Sclerrotium rolfiistrains* the particles demonstrated significant antifungal action. Tests were performed using the disc diffusion technique on biogenic ZnO NPs made from *Murraya koeniggi* and *Azadirachta indica* (L.) leaf extract (Elumalai et al., 2015). According to the findings, both ZnO NPs have significant antifungal activity. *Candida albicans* ATCC 2091, *Candida glabrata* NCIM3448, and *Cryptococcus* neoformans ATCC34664 were evaluated against green generated ZnO NPs using *Ziziphus nummularia* leaf extract by the minimum inhibitory concentration and time-kill assays, respectively (Padalia and Chanda, 2017). *C. albicans, C. glabrata*, and *C. neoformans* had MIC values of 1.25 mg/ml and 10 mg/ml, respectively. In the presence of *Candida albicans, Pongamia pinnata* seed extract-mediated ZnO NPs showed significant biofilm suppression efficacy at 50 mg/ml (Malaikozhundan et al., 2017). At a 100 μg/ml concentration, ZnO NPs produced using Cumin seed extract stopped 66% of *Rhizoctonia solani* fungus growth (Zare et al., 2017). Microwave aided the extraction of phytochemicals from Suaeda aegyptiaca to make ZnO NPs is a biogenic approach. The ZnO NPs generated by the former method had better inhibitory efficiency against *Candida albicans* and *Aspergillus oryzae* than those obtained by maceration. Increased concentration and irradiation power increased antifungal activity (Rajabi et al., 2017). *Aspergillus flavus*, *Aspergillus niger, Aspergillus fumigatus*, and *Fusarium solani* were evaluated against *Silybum marianum* extract-mediated ZnO nanoparticles. Every strain tested under 1 mg/ml showed significant inhibition (Hameed et al., 2019).

### Miscellaneous

Antibacterial activity against *E. coli, Klebsiella pneumonia, Salmonella choleraesuis*, and *Pseudomonas aeruginosa* has been shown by Au-Pt NPs composites of between 2 and 3 nm in size. (Zhao et al., 2014). According to recent investigations, bacterial growth inhibition has been linked to ATP synthesis and mitochondrial membrane potential. Antibacterial activity against *P. aeruginosa* was also studied using polyvinylpyrrolidone-coated PtNPs of various shapes and sizes, ranging from 2 to 20 nm. The toxicity of nanoparticles depends on their size, as shown by the toxic effects of NPs less than 3 nm on *P. aeruginosa*, whereas NPs larger than 3 nm had no or reduced damaging impact (Gopal et al., 2013). *E. coli* and *S. aureus* were both killed by PtNPs coated in PVP. *E. coli* growth was suppressed by small-sized NPs, consistent with findings from prior research (Taglietti et al., 2012). Antibacterial activity against *E. coli* in the vicinity of the metal composition, the rGO matrix, and the bacteria was improved by beading Pt/Ag NPs on reduced graphene oxide (rGO) nanosheets containing holes. The bacteria on the porous rGO matrix may be tracked using Ag ions released from the nanocomposite directly and quickly. Antibacterial activity against *E. coli, Lactococcus lactis*, and *Klebsiella pneumonia* was maximized when polyvinylpyrrolidone (PVP) nanoparticles (PVP/PtNPs) were combined as nanocomposites (Ramkumar et al., 2017).

## Recyclability and Reusability of Green-Synthesized Nanoparticles

The areas of materials engineering and nanotechnology are increasingly concerned with sustainability techniques, frameworks, and metrics in an attempt to mitigate environmental and health concerns connected with the manufacturing, use, and disposal of innovative nanomaterials (Dhingra et al., 2010). Veisi et al., synthesized Ag nanoparticles based on Thymbra spicata, the plant being rich source of thymol, carvacrol and myrcene (Veisi et al., 2018). In spite of the significant catalytic activity of Ag Nanoparticles/Thymbra. When Ag NPs/Thymbra were separated and reapplied in RhB and MB colour degradation, their recycling efficiency was determined correspondingly. No significant catalytic activity loss was seen when Ag NPs/Thymbra was recycled eight times for both dyes and 4-nitrophenol, indicating the great stability of Ag NPs/Thymbra. Moradnia et al., studied the synthesis of MgFeCrO_4_ based nanoparticles, using Tragacanth gel for the bioremediation of DB122 dye (Moradnia et al., 2020). Nanoparticles degraded DB122 dye with in 60 s and kinetics were compling with the pseudo first order kinetics. The photocatalyst was able to degrade the dye and didn’t showed any remarkable variation even after four runs. Nasrollahzadeh et al., studied the green synthesis of Palladium nanoparticles using barberry fruit extract and immobilized on reduced graphene oxide (Nasrollahzadeh et al., 2016). The reusability was tested on reduction of 4-nitrophenol with NaBH_4_ as model. The catalyst was recovered successfully after completion of activity without significance loss of its reducing activity. Similarly, researchers developed copper nanoparticles using Commersonia bartramia extract and immobilized using Al_2_O_3_ surface (Nasrollahzadeh et al., 2019). The catalyst was able to show significant changes upto 7th cycle, for reduction of 2,4-dinitrophenylhydrazine.

## Biocompatibility and Toxicity

The possible toxicity of nanoparticles to biological creatures has sparked controversy. Although pollens, fine sand and dust, volcanic ash, ocean spray, and biological material like viruses have been present in humans and other organisms for a long time, worry about nanoparticles is relatively new, due mostly to anthropogenic, synthetic, and manufactured nanoparticles. The skin, lungs, and gastrointestinal system are all often exposed to the outside world, making them ideal entrance points for nanomaterials of any type. Skin, on the other hand, is rather impermeable, making it less susceptible to NPs than the lungs and gastrointestinal system. Post-entry NPs may move from the entry ports into the circulatory and lymphatic systems and eventually into the tissues and organs of the human body. The worry is with a select kind of nanoparticles that induce permanent cell damage through oxidative stress or organelle injury, despite the fact that many nanomaterials are harmless and even helpful to health.

The size and content of the NPs seem to have a significant impact on the degree of harm they cause. Surface area, chemistry, and coating and functionalization of metallic nanoparticles are only a few of the many aspects that might have an adverse influence on the health of those exposed to them. In order to gather correct information for future policy and regulatory procedures, it is vital to focus on the research of toxicology of each item (Das R. K. et al., 2017). Due to the difficulty in evaluating nanoparticles’ toxicities using conventional toxicology methodologies, the toxicity evaluation of nanomaterials is difficult. In part, this is owing to nanomaterials’ tiny size and distinctive surface characteristics, which make them behave differently from bulk materials. Existing exposure pathways may be widened due to their modest size. In terms of toxicokinetics, nanomaterial surface qualities vary from those of bulk materials. Because nanoparticles have a large surface area, estimates based on mass are generally incorrect. As a result, it is difficult to collect accurate and repeatable exposure and toxicity data owing to the lack of reliable physical data such as surface area, composition, surface characteristics, and aggregation state (Hulla et al., 2015). The biological activity of nanoparticles generated with various reducing agents has been reported. Toxicity levels may vary dramatically depending on the biological agent utilized to synthesize biogenic nanoparticles, which are more common than non-biogenic ones. Nanoparticle characteristics are influenced by a variety of parameters, including their composition, size, shape, surface charge, and capping molecules (Venil et al., 2016). When tested against the MCF-7 breast cancer cell line, the cytotoxicity of silver nanoparticles generated using flexirubin was substantially greater than that of AgNPs made chemically. With 6-carboxypullulan and pullulan, it was found that AgNPs generated with 6-carboxypullulan were smaller than those synthesised with pullulan. Antimicrobial activity of carboxypullulan-mediated AgNPs was enhanced due of their larger negative zeta potential values (Coseri et al., 2015). AgNPs made from green tea extract (GT-AgNP) and coffee extract (CAgNP) demonstrated outstanding antibacterial effects in another fascinating investigation. For this reason, antimicrobial chemotherapy cannot employ C-AgNPs as they are hazardous to mammalian cells, while GT-AgNPs are benign (Rónavári et al., 2017). Standardized guidelines have also been developed recently in this regard. Researchers in the United Kingdom and the United States have made reference materials for nanotoxicity testing accessible to the public. For the first time, the International Alliance for Nano Environment, Human Health and Safety Harmonization began producing test methods for nanotoxicity testing. High-throughput nanomaterial screening seems promising and not too far away in view of the toxicity testing in the twenty-first century recommended by the US National Research Council (NRC).

## Challenges and Future Directions

Since the PK, biodistribution, and safety of nanoparticle-based therapies are heavily influenced by particle dimensions, the size and distribution of nanoparticles is generally acknowledged as a distinguishing property. The renal excretion of nanoparticles less than 20–30 nm is fast, while the mononuclear-phagocytic system (MPS; also known as reticuloendothelial systems) in the liver, spleen, and bone marrow is more effective in absorbing particles larger than 200 nm after administration (Moghimi et al., 2001) The liver and spleen are the primary sites of nanoparticles 150–300 nm in size (Gaumet et al., 2008), while colloids 200–400 nm in size are rapidly cleared by the liver (Douglas et al., 1987). Nanoparticles having a diameter of less than 200 nm may take advantage of the EPR effect for improved drug accumulation in tumours since tumour blood arteries have fenestrations ranging from 0.2 to 1.2 μm. When constructing a nanomedicine, the distribution of particle sizes must also be taken into consideration. While nanoparticles may theoretically have a wide range of sizes, if most of the particles are less than 200 nm in diameter, they may not have the full “benefits” associated with nanomedicine. The size and distribution of nanoparticles must be carefully managed throughout small-scale preparation and especially during larger-scale manufacture.

For nanoparticle behaviour and interaction with proteins and cells, surface characteristics of nanoparticles are crucial (Moghimi, 2003). For nanoparticles to remain stable and opsonize, several surface properties (such as charge, hydrophobicity, functional groups) must be present (Moghimi et al., 2001; Moghimi and Szebeni, 2003). These nanoparticles are coated with a variety of blood components in the process of opsonization; this triggers the complement pathway so that macrophages may remove them from circulation and prevent them from spreading (Gigli and Nelson, 1968). The biological synthesis of nanoparticles is a simple, low-risk method for producing them. The use of fungus to synthesise nanoparticles has been shown in a variety of domains. Smart medication delivery systems that deliver pharmaceuticals to the exact location where they are needed will aid in the early detection of sickness. Developing a biosensor and detection system that can protect crops from insects and diseases is a worthwhile endeavor.

In a nutshell, work on myconanotechnology is ongoing. The use of nanoparticles will continue to expand, but we must first investigate their toxicity, environmental buildup, and impact on human and animal health. Nanoparticles may also be utilized to cure a variety of serious ailments and open up new avenues in the biomedical area in the future, according to recent research. AgNPs might be used to power gadgets, which could help alleviate the current energy shortage. Nanoparticles have been extensively studied *in vitro*, but there is a dearth of data on their effects *in vivo*, as shown by the literature. There is still a lot of work that needs to be done in the subject of nanomaterials in order to make significant advancements across a variety of industries. However, despite the many advantages of using fungal-mediated metal nanoparticles, there are still a number of issues that must be addressed in order for this technology to become a reality on the market today.

Controlling nanostructure dispersity, which strongly influences electrical and optical characteristics, and isolating and purifying plural form are major issues in microbial nanobiosynthesis. The size distribution of a nanoparticle population is a critical feature that affects the particle’s behaviour in fluids. Methods including freeze-thawing, osmotic stress, and centrifugation may modify nanoparticle structures and cause aggregation and precipitation. Using appropriate methodologies might enhance microbial nanoparticle biosynthesis. The application of genetic engineering techniques and suitable microbial strains might assist overcome the disadvantages of slower production rate and polidispersity (relative to chemical-based nanomanufacturing) (Jeevanandam et al., 2018). A capping layer of biomolecules adsorbed on the surface of microbial biosynthetic nanoparticles acts as a stabilising agent and biological active layer (Ramya et al., 2015). The ability to identify capping agents (primarily peptides like glutathione, metallothioneins, membrane associated proteins, etc.) and purify nanoparticles (Jeevanandam et al., 2018) is critical for future *in vivo* medicinal applications.

## Conclusion

Many efforts have developed novel green synthesis processes during the previous several decades. Living creatures have a lot of potential for making nanomaterials that may be used in various sectors, including biomedicine. To manufacture nano-objects of the appropriate size and form, organisms ranging from primary bacteria to very sophisticated eukaryotes may all be employed. Prokaryotes are the most basic biomasses, making them easier to genetically alter to create more desirable synthesis chemicals. However, bacterial culture and large-scale manufacturing remain difficult compared to alternative methods. Bacteria were investigated as the first nano-factories to manufacture noble metal nanoparticles as a first step. The poor synthesis rate and restricted number of size and form distributions accessible, on the other hand, steered the research towards fungi and algae.

Fungi may be used to produce green nanoparticles on a massive scale. They’re simple to work with in downstream processing, and they release a lot of enzymes that help with the reduction. They also have metal filament tolerance, a high binding capacity, and intracellular uptake. However, eukaryotes have a considerably more difficult time genetically manipulating individual enzymes to increase production.

Plant extracts have lately been the subject of several studies, and the number of research papers published in this area has exploded in the past 2 years due to their widespread availability, environmental friendliness, and cost-effectiveness. This green chemistry strategy of utilizing living entities is in stark contrast with traditional chemical and physical processes that commonly involve hazardous compounds that have the potential to cause environmental toxicity, cytotoxicity, and carcinogenicity. Whilst biological entities have been extensively used to produce nanoparticles, the use of plant sources offers a straightforward, clean, non-toxic, and robust procedure that does not need any special culture preparation or isolation techniques that are normally required for bacteria and fungi-based techniques. In particular, the utilization of plant extracts for manufacturing nanoparticles is affordable, readily scaled up, and environment-friendly. Plant extracts have the ability to generate nanoparticles with a specified size, shape and content. Plant produced nanoparticles have the potential to be extensively employed in current medical processes utilizing nanoparticles such as fluorescent labelling in immunoassays, targeted administration of therapeutic medications, tumour death by heating (hyperthermia), and as antibacterial agents in bandages. On another front, plant produced nanoparticles have the potential to be exploited for the delivery of anti-microbiological chemicals for use as insecticides for agricultural crops. Moreover, agricultural crop wastes and food industry wastes are also ideal prospects for delivering supplies of plant-based bio-chemicals with the ability to synthesis metallic nanoparticles and related products. Despite the environmental advantages of using green chemistry based biological synthesis over traditional methods as discussed in this article there are some unresolved issues such as particle size and shape consistency, reproducibility of the synthesis process, and understanding of the mechanisms involved in producing metallic nanoparticles *via* biological entities. Therefore, there is a need for further research to analyze and comprehend the real biological synthesis dependent processes. This is a vastly untapped subject that needs much more research investment to properly leverage the green manufacturing of metallic nanoparticles through living entities.
