# Correction: Green metallic nanoparticles: biosynthesis to applications

**DOI:** 10.3389/fbioe.2026.1996246

**Published:** 2026-10-29

**Authors:** Hitesh Chopra, Shabana Bibi, Inderbir Singh, Mohammad Mehedi Hasan, Muhammad Saad Khan, Qudsia Yousafi, Atif Amin Baig, Md. Mominur Rahman, Fahadul Islam, Talha Bin Emran, Simona Cavalu

**Affiliations:** 1 Chitkara College of Pharmacy, Chitkara University, Rajpura, India; 2 Yunnan Herbal Laboratory, College of Ecology and Environmental Sciences, Yunnan University, Kunming, China; 3 The International Joint Research Center for Sustainable Utilization of Cordyceps Bioresources in China and Southeast Asia, Yunnan University, Kunming, China; 4 Department of Biochemistry and Molecular Biology, Faculty of Life Science, Mawlana Bhashani Science and Technology University, Tangail, Bangladesh; 5 Department of Biosciences, COMSATS University Islamabad, Sahiwal, Pakistan; 6 Unit of Biochemistry, Faculty of Medicine, University Sultan Zainal Abidin, Kuala Terengganu, Malaysia; 7 Department of Pharmacy, Faculty of Allied Health Sciences, Daffodil International University, Dhaka, Bangladesh; 8 Department of Pharmacy, BGC Trust University Bangladesh, Chittagong, Bangladesh; 9 Faculty of Medicine and Pharmacy, University of Oradea, Oradea, Romania

**Keywords:** nanoparticle, green nanotechnology, preparation, synthesis, application

In the published article, there were errors in the Introduction. The phrase “especially in the development of the Universe” was incorrect; the intended meaning was the development of nanoscale materials. The phrase “via a collection of fundamentals” was imprecise; the intended meaning was the foundational principles of green chemistry. The term “disruptive technologies” was also imprecise and has been replaced by “breakthrough technologies.”

A correction has been made to the section INTRODUCTION, paragraph 1. The corrected paragraph reads:

“There are tens of thousands of these goods on the market, most found in daily personal care, cosmetics, and apparel. Commercializing successful breakthrough technologies is essential for a wide range of human applications and worldwide progress, but critical attention is required in the materials’ potential, health evaluation, and environmental impact. There’s little doubt that nanoparticles (NPs) provide a health concern that has to be handled quickly, and their production and use are essentially unregulated, especially in the development of nanoscale materials. While new chemical processes are designed with little risk in mind, hazardous compounds are minimized or eliminated by applying the foundational principles of green chemistry. This is a crucial feature of the green chemistry developing industry (Hassan et al., 2021).”

In the published article, a parenthetical list was inadvertently duplicated in the Introduction. The sentence read: “Green production of metallic nanoparticles has been utilized to accommodate diverse biological components (e.g., bacteria, fungus, algae, and plant extracts) (e.g., bacteria, fungi, algae, and plant extracts).” The duplicate has been removed and the terminology standardized.

A correction has been made to the section INTRODUCTION, paragraph 2. The corrected sentence reads:

“Green production of metallic nanoparticles has been utilized to accommodate diverse biological components (e.g., bacteria, fungi, algae, and plant extracts).”

In the published article, the terms “living things” and “living creature” were used imprecisely in place of “living organisms” and “living organism.”

A correction has been made to the section GREEN SYNTHESIS OF NANOPARTICLES, paragraph 1. The corrected sentences read:

“Because of their enticing properties, many living organisms have effective biological factories, minimizing pollution while also recovering metals from industrial waste.”

“The capacity of a living organism to employ its metabolic processes to transform inorganic metallic ions into metal nanoparticles has opened the door to a relatively new and primarily untapped area of study (Baker et al., 2013).”

In the published article, water was described as a “reducing solvent.” Water is the solvent in this context and is not the reducing agent; the reduction is carried out by the plant-derived phytochemicals.

A correction has been made to the section GREEN SYNTHESIS OF NANOPARTICLES, subsection Biosynthesis of Nanoparticles Using Plants, paragraph 1. The corrected sentence reads:

“Using water as the solvent to synthesize nanoparticles has several benefits, including biocompatibility, scalability, and medicinal application (Noruzi, 2015).”

In the published article, the subsection heading “Zinc Nanoparticles” did not capture the scope of the compounds discussed, which are zinc oxide nanoparticles (ZnO NPs) rather than zinc metal nanoparticles. The heading has been updated for scientific precision.

A correction has been made to the section APPLICATIONS OF BIOSYNTHESIZED METALLIC NANOPARTICLES, subsection heading. The corrected heading reads: “Zinc-Based Nanoparticles.”

The reference for (KSV, 2017) was erroneously written as “Ksv, G. (2017). Green Synthesis of Iron Nanoparticles Using Green Tea Leaves Extract. J. Nanomedine. Biotherapeutic Discov. 07, 1. doi:10.4172/2155-983x.1000151”. It should be “Gottikumala, K. S. V. (2017). Green Synthesis of Iron Nanoparticles Using Green Tea Leaves Extract. J. Nanomedine. Biotherapeutic Discov. 07, 1. doi:10.4172/2155-983x.1000151”. The in-text citation in the section GREEN SYNTHESIS OF NANOPARTICLES, subsection Biosynthesis of Nanoparticles Using Plants, has been corrected accordingly from (KSV, 2017) to (Gottikumala, 2017).

In the published article, an incorrect reference was cited. The statement “Since discovering microbes’ ability to interact with, remove, and gather metallic elements from their surroundings, several biotechnological applications, such as bioremediation and bioleaching (Vicas et al., 2019) have been developed” cited Vicas et al. (2019), which reports the effect of nanoselenium particles on broccoli sprouts and does not concern microbial bioremediation or bioleaching. The citation has been removed and no replacement reference has been added.

A correction has been made to the section GREEN SYNTHESIS OF NANOPARTICLES, paragraph 1. The corrected sentence reads: “Since discovering microbes’ ability to interact with, remove, and gather metallic elements from their surroundings, several biotechnological applications, such as bioremediation and bioleaching have been developed.”

The reference “Vicas, S. I., Cavalu, S., Laslo, V., Tocai, M., Costea, T. O., and Moldovan, L. (2019). Growth, Photosynthetic Pigments, Phenolic, Glucosinolates Content and Antioxidant Capacity of Broccoli Sprouts in Response to Nanoselenium Particles Supply. Not Bot. Horti Agrobo 47, 1. doi:10.15835/nbha47311490” is no longer cited and has been removed from the reference list.

The original version of this article has been updated.

